# QM-CSA: A Novel Quantum Mechanics-Based Protocol for Evaluation of the Carcinogen-Scavenging Activity of Polyphenolic Compounds

**DOI:** 10.3390/foods13172708

**Published:** 2024-08-27

**Authors:** Veronika Furlan, Jelena Tošović, Urban Bren

**Affiliations:** 1 Faculty of Chemistry and Chemical Engineering, University of Maribor, Smetanova 17, SI-2000 Maribor, Slovenia; veronika.furlan@um.si; 2 Institute of Environmental Protection and Sensors, Beloruska Ulica 7, SI-2000 Maribor, Slovenia; 3 Faculty of Mathematics, Natural Sciences and Information Technologies, University of Primorska, Glagoljaška 8, SI-6000 Koper, Slovenia

**Keywords:** activation-free energies, chemical carcinogens, polyphenols, guanine, glutathione, quantum mechanical calculations, M11-L and MN12-L DFT functionals

## Abstract

In this study, a novel quantum mechanics-based protocol for the evaluation of carcinogen-scavenging activity (QM-CSA) is developed. The QM-CSA protocol represents a universal and quantitative approach to evaluate and compare the activation-free energies for alkylation reactions between individual polyphenolic compounds and chemical carcinogens of the epoxy type at physiological conditions by applying two scales: the absolute scale allowing for the comparison with guanine and the relative scale allowing the comparison with glutathione as a reference compound. The devised quantum mechanical methodology was validated by comparing the activation-free energies calculated with 14 DFT functionals in conjunction with two implicit solvation models (SMD and CPCM) and the experimental activation-free energies for reactions between nine investigated chemical carcinogens and guanine. According to the obtained results, the best agreement with experimental data was achieved by applying DFT functionals M11-L and MN12-L in conjunction with the flexible 6-311++G(d,p) basis set and implicit solvation model SMD, and the obtained uncertainties were proven to be similar to the experimental ones. To demonstrate the applicability of the QM-CSA protocol, functionals M11-L, and MN12-L in conjunction with the SMD implicit solvation model were applied to calculate activation-free energies for the reactions of nine investigated chemical carcinogens of the epoxy type with three catechins, namely EGCG, EGC, and (+)-catechin. The order of CSA in this series of catechins in comparison to guanine and glutathione was determined as (+)-catechin > EGC > EGCG. The obtained results, for the first time, demonstrated the evaluation and comparison of CSA in a series of selected catechins with respect to glutathione and guanine. Moreover, the presented results provide valuable insights into the reaction mechanisms and configurations of the corresponding transition states. The novel QM-CSA protocol is also expected to expand the kinetic data for alkylation reactions between various polyphenolic compounds and chemical carcinogens of the epoxy type, which is currently lacking in the scientific literature.

## 1. Introduction

**Figure 1:**
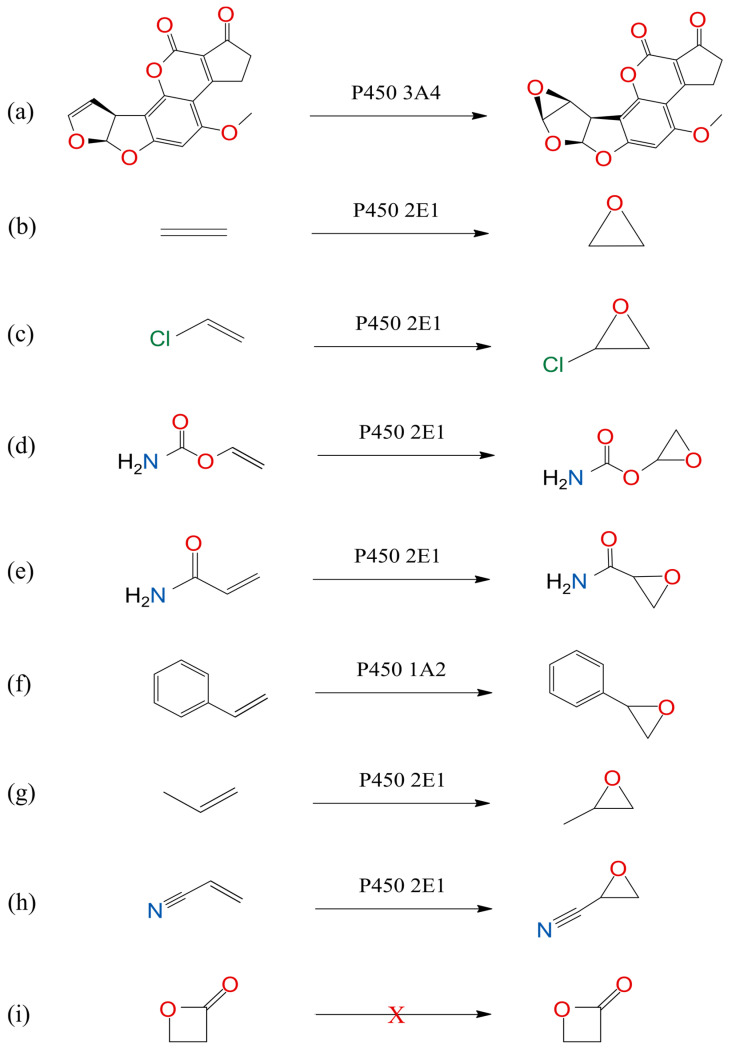
Biotransformations of nine procarcinogens into investigated genotoxic ultimate chemical carcinogens, among which eight require metabolic activation by cytochrome P450 enzymes: (**a**) aflatoxin B1 exo-8,9-epoxide, (**b**) ethylene oxide, (**c**) chloroethylene oxide, (**d**) vinyl carbamate epoxide, (**e**) glycidamide, (**f**) styrene oxide, (**g**) propylene oxide, and (**h**) 2-cyanoethylene oxide, while (**i**) beta-propiolactone needs no metabolic activation by cytochrome P450 enzymes and acts as a genotoxic chemical carcinogen by itself.

Cancer represents the second deadliest disease of the modern era after cardiovascular disorders. Carcinogenesis is usually triggered by DNA damage, which is, in most cases, caused by genotoxic chemical carcinogens that enter our body from the environment or are ingested with food. The term chemical carcinogens refers to compounds that contribute to cancer initiation by causing damage or mutations in DNA. If DNA damage results in oncogene activation or the deactivation of tumor suppressor genes, normal cells become neoplastic, which leads to cancer development [1]. The current study is focused on nine exogenous chemical carcinogens, which are implicated in the etiology of various cancer types. Aflatoxin B1 represents the most potent natural chemical carcinogen known to man, synthesized by molds of the *Aspergillus* genus, which grow on cereals, nuts, and oils in storage units with high temperatures and humidity. Aflatoxin B1 is, after ingestion, metabolized by cytochrome P450 3A enzymes to the highly reactive ultimate chemical carcinogen aflatoxin B1-exo-8,9-epoxide (AFB1), which can damage DNA and initiate cancer development [2]. Ethylene oxide (EO) is the ultimate chemical carcinogen of ethylene and is used for the sterilization of medical instruments in third-world countries [3]. Chloroethylene oxide (CEO) represents an electrophilic metabolite of vinyl chloride, which is used in the farming, plastic, and electrical industries [4]. Vinyl carbamate epoxide (VCE) is the ultimate chemical carcinogen of urethane, which is formed as a by-product of fermentation [5]. The ultimate chemical carcinogen glycidamide (GA) represents a metabolite of acrylamide, which develops while frying and baking various foods [6]. Styrene oxide (SO) is metabolized from styrene, which is often applied in the plastic industry [7]. Propylene oxide (PO) is the ultimate chemical carcinogen of propylene used as a medium for sterilization as well as for the production of polyurethane foams, solvents, and coolants [8]. 2-Cyanoethylene oxide (CyEO) is formed by the metabolic oxidation of acrylonitrile, which is applied to the manufacturing of acrylic fibers, nitrile rubber, and adhesives [9]. Finally, beta-propiolactone (BPL) represents a chemical carcinogen used in vaccines to prevent viral infections [10]. Biotransformations of nine procarcinogens into investigated genotoxic ultimate chemical carcinogens are depicted in Figure 1. Except for beta-propiolactone, the investigated chemical carcinogens are metabolized in the human body by cytochrome P450 enzymes into genotoxic ultimate chemical carcinogens of the epoxy type, which react with DNA primarily at the N7 atom of the most nucleophilic base guanine, leading to DNA damage and cancer initiation [2,4].

Glutathione represents a cellular tripeptide and an important hydrophilic biomolecule that participates in cellular detoxification processes. Glutathione regulates oxidative stress and protects cells from lipid peroxides, reactive oxygen and nitrogen species, and electrophilic xenobiotics [11]. Glutathione acts as the strongest natural non-enzymatic scavenger of chemical carcinogens in human cells by reducing their reactivity and subsequent toxicity. The conjugation of chemical carcinogens with glutathione facilitates their greater polarity and easier elimination from the body. According to recent studies, glutathione also represents the key regulator of cancer cell differentiation, proliferation, apoptosis, and immune function [12].

Polyphenols form a large group of more than 10,000 secondary plant metabolites with one or more aromatic rings containing one or more hydroxyl groups. Polyphenols are abundantly present in a majority of fruits, vegetables, and herbs and exert several beneficial health effects [13,14]. According to their chemical structure, polyphenols can be divided into five classes, namely flavonoids, phenolic acids, stilbenes, lignans, and tannins, among which flavonoids and polyphenolic acids are the most abundant [15]. Catechins (flavan-3-ols) represent one of the most important subclasses of flavonoids, gaining recognition as chemopreventive agents due to their various beneficial health effects as well as their safety [16]. Green tea (*Camellia sinensis*) represents one of the most consumed beverages, which is a rich source of epistructured gallocatechins, namely epigallocatechin-3-gallate (EGCG) and epigallocatechin (EGC), with proven health benefits [17,18,19]. On the other hand, cocoa represents a rich source of nonepistructured catechins, especially (+)-catechin, exerting various beneficial biological effects [20]. Several in vitro and in vivo studies reported that green tea and cocoa consumption exhibits antioxidative and anti-inflammatory effects, lowers the risk of hypertension, prevents cardiovascular disease and type II diabetes, aa well as improves brain function, although catechins are mainly investigated for their cancer chemopreventive and chemotherapeutic effects [21,22,23,24,25].

EGCG and EGC represent important catechins obtained from green tea with various known beneficial health effects, including antioxidant [26,27], anti-inflammatory [28], antimicrobial [29], and anticarcinogenic [30] effects. EGCG and EGC exhibited inhibitory activities on cyclooxygenase 2 (COX-2), leading to suppressed cell proliferation, tumor invasion, and anti-inflammatory effects [31,32]. The strong anticarcinogenic character of the green tea catechins EGCG and ECG was demonstrated through the reduction of carcinogen-induced reactive oxygen species (ROS), antiproliferative, and pro-apoptotic effects in MCF7 breast cancer cells [33]. The most prevalent green tea catechin, EGCG, also exhibited antiangiogenic and antimetastatic effects as well as suppressed proliferation and invasion of cancer cells through down-regulation of matrix metalloproteinase (MMP) enzymes MMP-2 [34] and MMP-9 [35]. EGCG also inhibited the activation of hypoxia-inducible factor-1 (HIF-1α), nuclear factor kappa B (NF-κB), and vascular endothelial growth factor (VEGF) expression, resulting in the suppression of angiogenesis and breast cancer progression [36]. Moreover, EGCG also demonstrated inhibition of B-cell lymphoma 2 (Bcl-2) and NF-κB in hepatocellular cancer cells LM6 [37], and deactivated activator protein-1 (AP-1) expression, resulting in apoptosis of AGS gastric cancer cells [38].

Anticarcinogenic effects of important catechin from cocoa (+)-catechin have also been extensively investigated [39,40,41,42]. (+)-Catechin exhibited anticancer effects against A549 lung cancer cells through the dose-dependent induction of cyclin kinase inhibitor p21 and suppression of cyclin E1, leading to cell cycle arrest [42]. It was also reported that (+)-catechin up-regulated p53 in colorectal cancer cells HT-29, promoting cell cycle arrest and inhibition of cell proliferation. Moreover, (+)-catechin demonstrated cytotoxicity in colorectal cancer cells HT-29 and activated their apoptosis through up-regulation of caspases 3 and 8 [41]. (+)-Catechin can also induce the inhibition of angiogenesis by regulating nitric oxide (NO) and tumor necrosis factor alpha (TNF-α) production [39]. Furthermore, (+)-catechin reduced VEGF mRNA levels in B16F-10 melanoma cells. The anticancer effects of (+)-catechin against human colon adenocarcinoma cells HCT 15 and HCT 116 as well as against human larynx carcinoma HepG-2 cells were also investigated [40]. The obtained results showed that (+)-catechin treatment resulted in decreased cell proliferation and increased the presence of apoptotic bodies in the investigated cancer cells.

**Figure 2:**
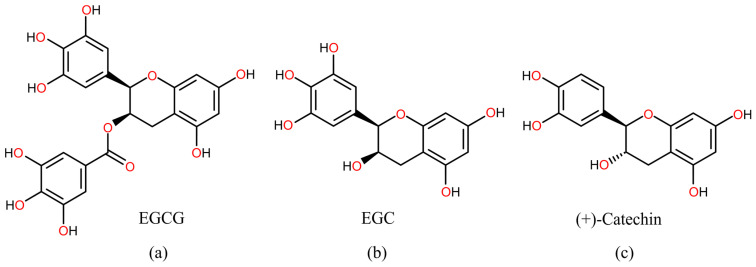
Structural formulas of (**a**) epigallocatechin-3-gallate (EGCG), (**b**) epigallocatechin (EGC), and (**c**) (+)-catechin.

Although the anticarcinogenic effects of catechins EGCG, EGC, and (+)-catechin (Figure 2) were reported in several experimental studies, their potential as direct scavengers of investigated chemical carcinogens remains unexplored.

Nowadays, it is possible to perform quantum mechanical calculations and obtain reliable kinetic data for small- or medium-sized systems up to 100 atoms at a reasonable computational cost. Quantum mechanics-based methodologies currently play an important role in investigations of reaction mechanisms and represent promising alternatives to experimental approaches, particularly in the case of difficult reaction conditions and expensive or (geno)toxic chemicals. In our previous studies, we reported that the polyphenols 6-gingerol and xanthohumol and its derivatives isoxhanthohumol, 8-prenylnaringenin, and 6-prenylnaringenin represent potential scavengers of ultimate chemical carcinogens of the epoxy type [43,44]. The obtained results at the Hertree–Fock/6-311++G(d,p) level of theory, in combination with two implicit solvation models, confirmed the proposed *S_N_2* reaction mechanism of the most abundant monoanionic forms of the studied polyphenols at physiological conditions. Taking advantage of quantum mechanical methodologies to systematically investigate the carcinogen-scavenging activity (CSA) of polyphenolic compounds, therefore, represents a safe, fast, and inexpensive alternative to experimental assays.

It must be noted that plant extracts contain a complex composition of various polyphenolic compounds, which results in challenging chemical analyses and exhaustive evaluations of compounds or combinations of compounds that are responsible for the specific biological activity [45]. By using quantum mechanics-based computational approaches, the potential of individual polyphenolic compounds, such as EGCG, EGC, and (+)-catechin, to scavenge chemical carcinogens can be quantitatively evaluated and compared. The main purpose of this comparison is to identify polyphenolic compounds with the highest CSA and hopefully guide and accelerate the design of efficient strategies to prevent cancer initiation. However, a universal and quantitative quantum mechanical methodology to evaluate and compare the carcinogen-scavenging activity of individual polyphenolic compounds by applying an appropriate combination of Density Functional Theory (DFT) functionals and implicit solvation models is still not established. The applicability of theoretical quantum mechanics-based approaches to study chemical reactions is mainly influenced by the selected method, flexible basis set, and implicit solvation model, which determine the accuracy of the obtained results. Moreover, the influence of the pH on the protonation state of polyphenolic compounds should also be taken into account. pKa values of hydroxyl groups of polyphenolic compounds determine the portion of neutral, mono-anionic, and dianionic forms of polyphenolic compounds at a specific pH value [46].

In this study, a novel quantum mechanics-based protocol for the evaluation of carcinogen-scavenging activity (QM-CSA) by applying the advanced Density Functional Theory (DFT) functionals M11-L and MN12-L in combination with the flexible 6-311++G(d,p) basis set and implicit solvation model density (SMD) is presented. The developed QM-CSA protocol allows for the evaluation of the molecular mechanisms and kinetics of the alkylation reactions involved in the CSA of polyphenolic compounds by applying two scales: the absolute scale yielding the comparison with guanine and the relative scale yielding the comparison with glutathione as a reference compound. To demonstrate the applicability of the devised QM-CSA protocol, the CSA of the important epicatechins EGCG and EGC, as well as that of nonepistructured (+)-catechin, was evaluated. The selected catechins have not been previously studied in the role of scavengers of chemical carcinogens; therefore, their mechanistic and kinetic data are provided for the first time.

## 2. Computational Methods

The aim of this study was to develop a standardized quantum mechanics-based methodology for the evaluation of carcinogen-scavenging activity (QM-CSA). The QM-CSA protocol is intended to establish a universal strategy to evaluate and compare the primary CSA of natural polyphenols by applying an appropriate combination of DFT functional, flexible basis set, and implicit solvation model. The QM-CSA protocol is based on kinetic evaluations and considers the biological conditions (e.g., pH or solvent) that may influence the CSA.

The QM-CSA protocol aims to provide:

1. The identification of the main molecular mechanism of alkylation reactions between nine chemical carcinogens and polyphenolic compounds at physiological conditions (pH 7.4 and an aqueous environment).
2. Two scales for the quantification of CSA: the absolute scale based on the comparison of the calculated activation-free energies for alkylation reactions of polyphenols with guanine and the relative scale using glutathione as a reference compound.
3. The establishment of the order of CSA in a series of polyphenolic compounds at physiological conditions.
4. The identification of the most effective scavengers of chemical carcinogens of the epoxy type and the structural features responsible for their activity.

The QM-CSA protocol has been devised to systematically address the important aspects, which are presented in the following subsections.

### 2.1. Investigation of Molecular Mechanisms of Alkylation Reactions of Guanine, Glutathione, and Polyphenolic Compounds with Chemical Carcinogens at Physiological Conditions

**Scheme A1:**
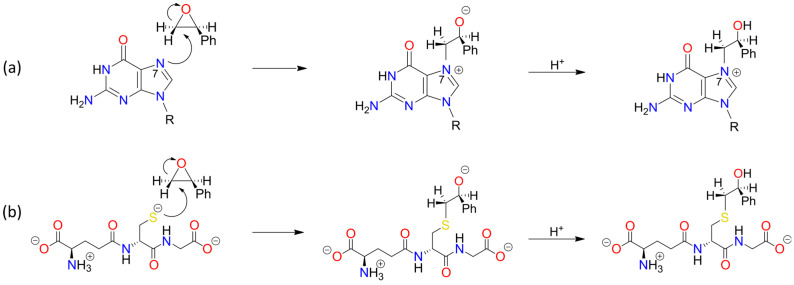
The proposed *S_N_2* mechanism for the reaction of styrene oxide with (**a**) guanine and (**b**) glutathione.

**Scheme A2:**
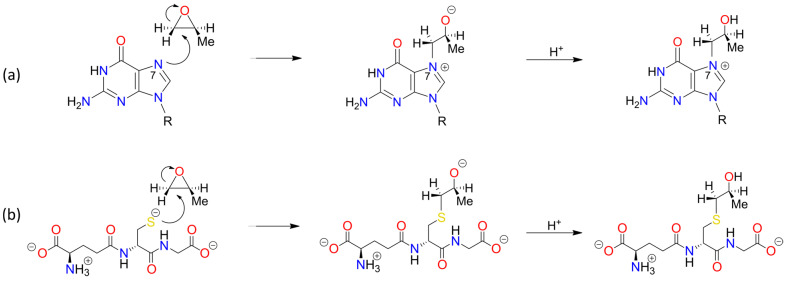
The proposed *S_N_2* mechanism for the reaction of propylene oxide with (**a**) guanine and (**b**) glutathione.

**Scheme A3:**
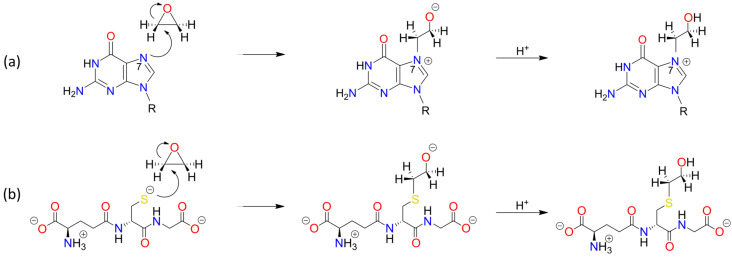
The proposed *S_N_2* mechanism for the reaction of ethylene oxide with (**a**) guanine and (**b**) glutathione.

**Scheme A4:**
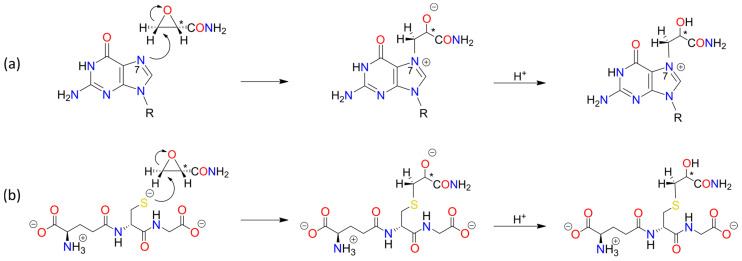
The proposed *S_N_2* mechanism for the reaction of glycidamide with (**a**) guanine and (**b**) glutathione. * represents the chiral center.

**Scheme A5:**
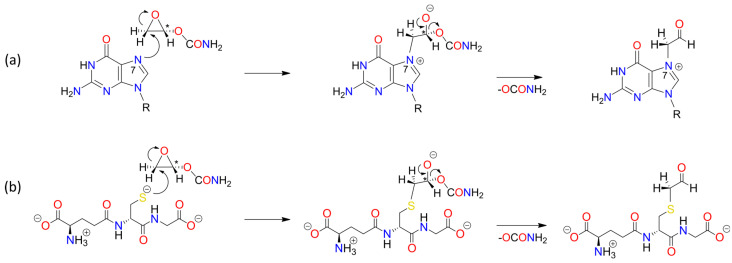
The proposed *S_N_2* mechanism for the reaction of vinyl carbamate epoxide with (**a**) guanine and (**b**) glutathione. * represents the chiral center.

**Scheme A6:**
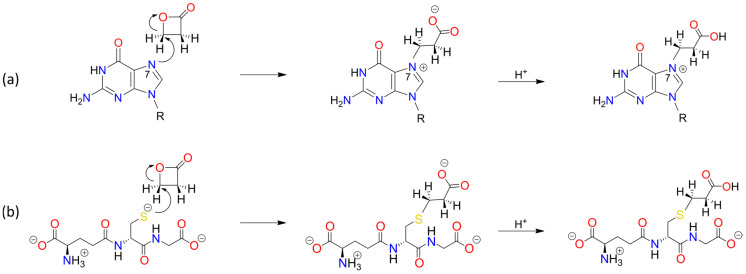
The proposed *S_N_2* mechanism for the reaction of beta-propiolactone with (**a**) guanine and (**b**) glutathione.

**Scheme A7:**
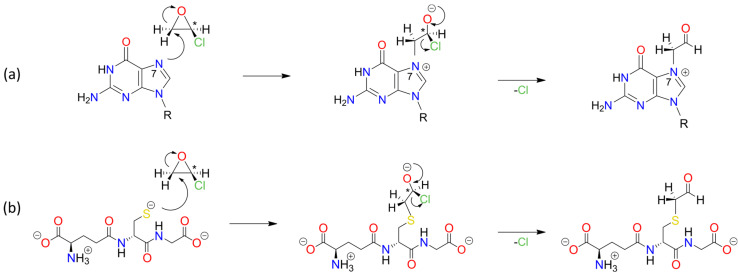
The proposed *S_N_2* mechanism for the reaction of chloroethylene oxide with (**a**) guanine and (**b**) glutathione. * represents the chiral center.

**Scheme A8:**
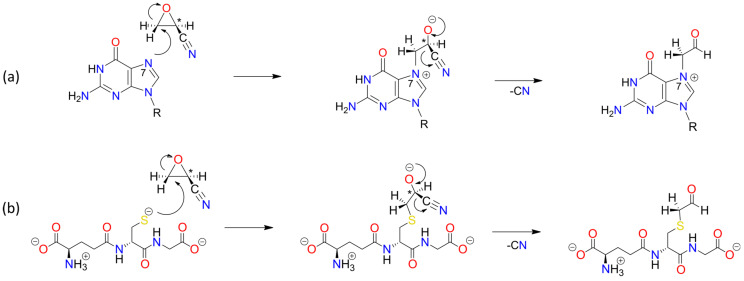
The proposed *S_N_2* mechanism for the reaction of 2-cyanoethylene oxide with (**a**) guanine and (**b**) glutathione. * represents the chiral center.

**Scheme A9:**
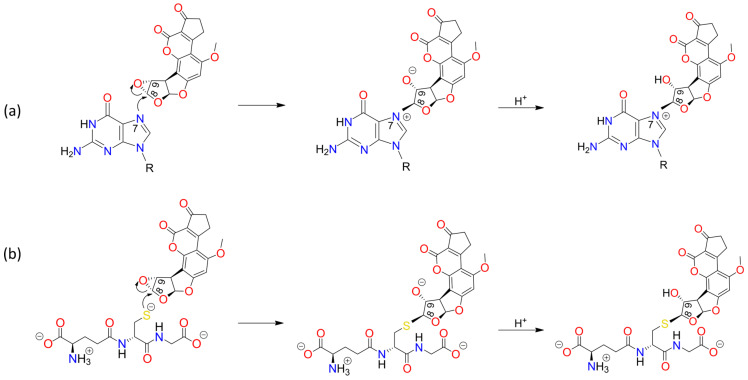
The proposed *S_N_2* mechanism for the reaction of AFB1 exo-8,9-epoxide with (**a**) guanine and (**b**) glutathione.

All nine studied chemical carcinogens of the epoxy type represent electrophilic species that primarily react with the most nucleophilic DNA base guanine at the atom N7, resulting in the covalent chemical carcinogen–guanine adduct formation. The rate-limiting step for the reactions of chemical carcinogens with nucleophilic DNA bases is the epoxy ring opening [6]. Using the ab initio Hertree–Fock method in combination with the flexible 6-311++G(d,p) basis set and self-consistent reaction field implicit solvation models, our research group successfully reproduced experimentally determined activation-free energies of alkylation reactions between the most reactive DNA base guanine and nine genotoxic chemical carcinogens, namely aflatoxin B1 exo-8,9-epoxide [2], 2-cyanoethylene oxide [9], glycidamide [6], chloroethylene oxide [4], ethylene oxide [3], styrene oxide [7], propylene oxide [8], beta-propiolactone [10], and vinyl carbamate epoxide [5], which corresponded to the proposed *S_N_2* reaction mechanism. Subsequent protonation is a fast process because of the proton-rich microenvironment surrounding the negatively charged DNA. The depurination of formed adducts then leads to mutations and chromosomal aberrations [6]. The proposed *S_N_2* substitution mechanisms for the reactions of nine investigated chemical carcinogens with (a) guanine and (b) glutathione are presented in Scheme A1, Scheme A2, Scheme A3, Scheme A4, Scheme A5, Scheme A6, Scheme A7, Scheme A8 and Scheme A9 in Appendix A, which were prepared in the ChemDraw program. For the rate-limiting step of alkylation reactions between polyphenolic compounds and the nine investigated chemical carcinogens at physiological conditions (pH 7.4 and an aqueous solution), the *S_N_2* reaction mechanism is proposed as well.

### 2.2. The Determination of an Appropriate Combination of Density Functional, Flexible Basis Set, and Implicit Solvation Model

In order to determine the best combination of quantum mechanical method, flexible basis set, and implicit solvation model to calculate activation-free energies of alkylation reactions between the nine chemical carcinogens and guanine, 14 advanced DFT functionals in combination with the 6-311++G(d,p) basis set and two implicit solvation models, namely the Conductor-like Polarizable Continuum Model (CPCM) and the Solvation Model Density (SMD), were applied. The selected set of DFT functionals included 10 frequently applied meta-Generalized Gradient Approximations (GGA) exchange–correlation functionals, namely M05-2X [47], M06-2X [48], M06-HF [49], M08-HX [50], M11 [51], M11-L [52], MN12-L [53], MN12-SX [54], MN15 [55], and MN15-L [56], 2 meta-Non-separable Generalized Gradient Approximations (NGA) exchange–correlation functionals N12 [57] and N12-SX [54], as well as 2 hybrid-GGA exchange–correlation functionals B3LYP-D3 [58] and WB97XD [59]. All quantum mechanical calculations of activation-free energies were performed with the program package Gaussian 16 on the supercomputer system HPC Vega located at the Institute of Information Science (IZUM). The computationally obtained activation-free energies for reactions between guanine and investigated chemical carcinogens were then compared to their corresponding experimental counterparts. The latter were determined from the experimental reaction rate constants k [2,3,4,5,6,7,8,9,10], which can be obtained by applying the Transition State Theory (TST) of Eyring (Equation (1)) [60]:

(1) $$k=\frac{k_{B}T}{h}e^{\left(-\frac{{\Delta G}^{‡}}{k_{B}T}\right)}$$

where $k_{B}$ and *h* are the Boltzmann and Planck constants, respectively; *T* is the temperature (K); and ${\Delta G}^{‡}$ is the activation-free energy, representing the free energy difference between the transition states and reactants (kcal/mol). The main advantage of using TST is that it requires only the free energy of reactants and transition state structures for the calculation of the corresponding ${\Delta G}^{‡}$, which allows for its application to various chemical reactions. TST is applicable for medium- to large-size systems, such as chemical carcinogens of the epoxy type reacting with guanine. To evaluate the accuracy of the devised QM-CSA methodology, the calculated activation-free energies for the reactions of the investigated chemical carcinogens with guanine were compared to their experimental counterparts.

Based on the analysis of the obtained results (Section 3), the combination of meta-GGA functional M11-L [52] or MN12-L [53] with flexible basis set 6–311++G(d,p) and SMD implicit solvation model provided the best agreement with experimental data. Meta-GGA exchange-correlation functional M11-L is based on a dual-range local exchange to ensure accuracy for single-configurational as well as multiconfigurational molecules, solid-state lattice constants, and barrier heights [52]. A local exchange-correlation meta-GGA functional MN12-L was designed to study transition metal chemistry, atomization energies, ionization potentials, noncovalent interactions, solid-state lattice constants, and barrier heights [53]. Both M11-L and MN12-L functionals have also been recommended for calculations of activation-free energies by their developers [52,53] and successfully applied by independent authors to predict reaction barrier heights [61,62,63].

In terms of the choice of a flexible basis set, it is crucial to include polarization and diffuse functions for nonhydrogen atoms. The application of diffuse functions is important to investigate polyphenolic compounds in anionic forms, which play a key role in CSA. The Gaussian basis set 6-311++G(d,p) represents the flexible basis set of choice in terms of more satisfactory results and lower computational cost when compared with the augmented correlation consistent polarized valence double zeta (aug-cc-pVDZ) basis set [64].

As the presence of the solvent significantly affects the reactivity of molecules, it is important to include solvation effects in quantum mechanical calculations of alkylation reactions between chemical carcinogens of the epoxy type and polyphenolic compounds. Different solvation models and their advantages were comprehensively addressed in review articles [65,66,67]. In this study, the most widely applied implicit solvation models, namely SMD and CPCM, were included in quantum mechanical calculations of activation-free energies to mimic the aqueous environment surrounding chemical carcinogens and guanine in human cells. The combination of DFT functionals M11-L or MN12-L with flexible 6-311++G(d,p) basis sets and the SMD implicit solvation model proved to be the most successful at reproducing the experimental activation-free energies. The implicit solvation model SMD employs the quantum mechanical charge density of a solute that interacts with a polarizable continuum of the solvent. SMD represents a universal implicit solvation model that can be applied to any charged or uncharged solute in various polar and organic solvents [67]. To conclude, DFT functionals M11-L and MN12-L in combination with the flexible 6-311++G(d,p) basis set and SMD solvation model are recommended to study alkylation reactions with chemical carcinogens of the epoxy type.

### 2.3. Determination of Anticarcinogenic Potential of Various Polyphenolic Compounds as Scavengers of Nine Investigated Chemical Carcinogens of the Epoxy Type Using QM-CSA Methodology

For comparison and evaluation of the carcinogen-scavenging activity of polyphenolic compounds, it is recommended to apply the same quantum mechanical methodology, namely DFT functional M11-L or MN12-L, in conjunction with the flexible 6-311++G(d,p) basis set and SMD implicit solvation model. It should also be noted that the reliability of the obtained results depends on the chosen functional, and that only functionals M11-L and MN12-L in combination with the SMD solvation model, proved to accurately reproduce the experimental activation-free energies for alkylation reactions of the nine studied chemical carcinogens with guanine. Moreover, the studied alkylation reactions should take place in an aqueous solution at a physiological pH in order to rightfully compare the calculated activation-free energies. By applying the same computational methodology, a comprehensive comparison of the CSA of different polyphenolic compounds can be performed by investigating from one to all nine chemical carcinogens, namely aflatoxin B1-exo-8,9-epoxide, ethylene oxide, chloroethylene oxide, vinyl carbamate epoxide, glycidamide, styrene oxide, propylene oxide, 2-cyanoethylene oxide, and beta-propiolactone.

In this study, the CSA of three investigated catechins, EGCG, EGC, and (+)-catechin, as well as glutathione under physiological conditions (pH = 7.4, aqueous solution), was determined by applying DFT functionals M11-L and MN12-L in combination with the flexible 6-311++G(d,p) basis set and SMD solvation model. EGCG, EGC, and (+)-catechin were studied in their nucleophilic (monoanionic) forms at physiological conditions. The pKa values of the most acidic protons of EGCG, EGC, and (+)-catechin at the pH of 7.4 were calculated with MarvinSketch software (20.19, ChemAxon, Budapest, Hungary) [68], and their values were in agreement with the experimentally determined ones [46]. Geometry optimization and vibrational analysis of reactant structures were performed to obtain the corresponding minima on the potential energy surface. To obtain the initial geometries of corresponding transition states, a relaxed potential surface scan was performed [35]. The identified initial geometries were subsequently subjected to geometry optimization and vibrational analysis [37]. Quantum mechanical calculations of activation-free energies were performed with Gaussian 16 on the supercomputer HPC Vega located at IZUM. Only the first step of the reaction between the individual chemical carcinogen and the investigated catechins, as well as guanine and glutathione, was examined, as subsequent protonation is believed to be a fast process due to a proton-rich microenvironment surrounding the product of the initial reaction step [43].

### 2.4. Evaluation of the CSA of Polyphenolic Compounds by Applying Two Scales

A comparison of different polyphenolic compounds in the role of scavengers of nine chemical carcinogens can be established by applying two scales.

**Figure 3:**
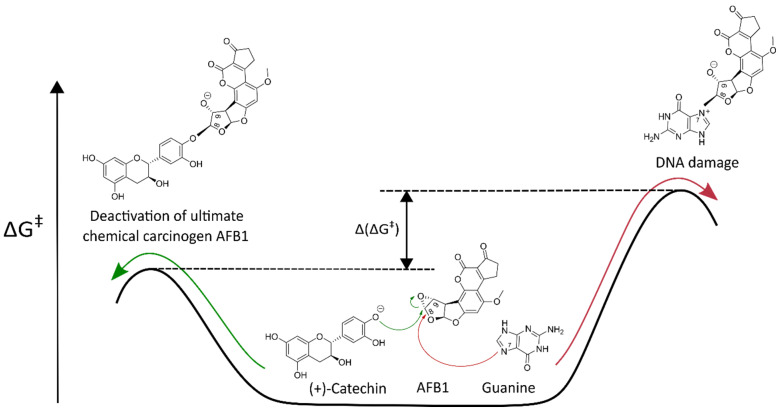
Activation-free energy, ${\Delta G}^{‡}$, for the reaction between the ultimate chemical carcinogen (AFB1 exo-8,9-epoxide) and its polyphenolic scavenger ((+)-catechin) (green arrow) in comparison with the activation-free energy, ${\Delta G}^{‡}$, for a competing reaction between the same chemical carcinogen and guanine (red arrow).

The first scale allows for an absolute comparison between activation-free energies ${\Delta G}^{‡}$ for alkylation reactions of the investigated polyphenol and the most nucleophilic DNA base, guanine, with the same chemical carcinogens. For this purpose, the calculated ${\Delta G}^{‡}$ value of the reaction between polyphenol and the chemical carcinogen is compared with the ${\Delta G}^{‡}$ value of the corresponding competing reaction between guanine and the same chemical carcinogen. If the ${\Delta G}^{‡}$ value for the reaction of the chemical carcinogen with polyphenol is lower than the ${\Delta G}^{‡}$ value of the competing reaction of the same chemical carcinogen with guanine, the investigated polyphenol represents an efficient scavenger of this chemical carcinogen (Figure 3). The lower ${\Delta G}^{‡}$ value directly corresponds to a higher reaction rate constant and indicates a higher CSA of the polyphenolic compound [43]. The absolute scale is, therefore, defined by Equation (2):

(2) $$\Delta \left({\Delta G}^{‡}\right)=\left(\Delta G_{polyphenol}^{‡}-\Delta G_{guanine}^{‡}\right)$$

In the second scale, glutathione, the strongest natural scavenger of chemical carcinogens in human cells, is applied as a reference compound [69,70]. Alkylation reactions between glutathione and the same nine chemical carcinogens were investigated under identical conditions (pH value of 7.4, aqueous solution) as the reactions with polyphenols and guanine by applying the DFT functionals M11-L and MN12-L in combination with the flexible 6-311++G(d,p) basis set and SMD solvation model. The greater or lower activation-free energies of the investigated polyphenols compared to glutathione determine their relative CSA. The formula to calculate the factor $r^{glutathione}$ is provided in Equation (3):

(3) $$r^{glutathione}=\frac{{\Delta G}_{polyphenol}^{‡}}{{\Delta G}_{glutathione}^{‡}}$$

where ${\Delta G}_{polyphenol}^{‡}$ represents the activation-free energy for the reaction of polyphenol with the chemical carcinogen, and ${\Delta G}_{glutathione}^{‡}$ represents the activation-free energy for the competing reaction of glutathione with the same chemical carcinogen. Therefore, the relative CSA of polyphenolic compounds can be expressed as r-times lower or higher than that of glutathione ($r^{glutathione}$ = 1). In this way, the most effective polyphenolic scavengers of chemical carcinogens can be identified based on the comparison with guanine and glutathione, and a ranked series of activity can also be established. This approach is also expected to maximize the cancelation of errors inherent to all calculated activation-free energies.

## 3. Results and Discussion

### 3.1. Validation of the Developed QM-CSA Protocol

**Figure 4:**
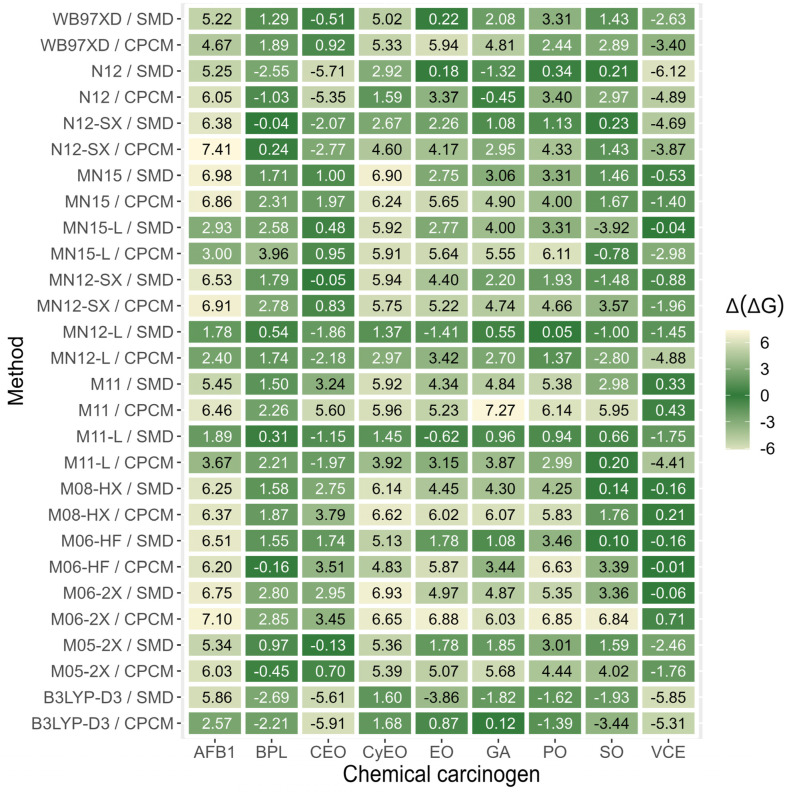
The heat map of differences $\Delta \left(\Delta G\right)=(\Delta G_{cal}^{‡}-\Delta G_{exp}^{‡})$ between the activation-free energies calculated with 14 DFT functionals in combination with the flexible 6-311++G(d,p) basis set and implicit solvation models CPCM or SMD ($\Delta G_{cal}^{‡}$), and the corresponding experimental activation-free energies ($\Delta G_{exp}^{‡}$) for reactions between the nine investigated chemical carcinogens and guanine. The dark green color designates a good agreement with the corresponding experimental activation-free energy for the alkylation reaction between individual chemical carcinogens and guanine (ideally $\Delta \left(\Delta G\right)=(\Delta G_{cal}^{‡}-\Delta G_{exp}^{‡})=0$), while the light green color designates a poor agreement with the corresponding experimental value ($\Delta \left(\Delta G\right)$>> 0 or $\Delta \left(\Delta G\right)$<< 0).

Due to the absence of experimental data on the activation-free energies of alkylation reactions between the nine studied chemical carcinogens and polyphenolic compounds, a test set of nine experimental activation-free energies for the competing reactions between the nine chemical carcinogens and guanine was used to validate the devised QM-CSA protocol. In Figure 4, the differences between the experimental activation-free energies and the ones calculated with 14 most frequently applied DFT functionals, namely M05-2X, M06-2X, M06-HF, M08-HX, M11, M11-L, MN12-L, MN12-SX, MN15, MN15-L, N12, N12-SX, B3LYP-D3, and wB97XD, in combination with the flexible 6-311++G(d,p) basis set and two implicit solvation models, namely SMD and CPCM, are collected. The dark green color designates the best agreement with the corresponding experimental activation-free energy for the alkylation reaction between individual chemical carcinogens and guanine, while the light green color designates a poor agreement with the corresponding experimental value.

As can be observed from Figure 4, a large majority of studied functionals accurately reproduced the experimental activation-free energy for the reactions between guanine and BPL with a minimum Δ(ΔG) difference obtained by the combination N12-SX/SMD (−0.04 kcal/mol) and a maximum Δ(ΔG) difference in the case of MN15-L/CPCM (3.96 kcal/mol). On the other hand, a large majority of the applied functionals very poorly reproduced the experimental activation-free energy in the case of AFB1, with a minimum Δ(ΔG) difference obtained with MN12-L/SMD (1.78 kcal/mol) and a maximum Δ(ΔG) difference in the case of MN15/SMD (6.98 kcal/mol). Interestingly, the most frequently applied hybrid meta-GGA functional M06-2X in combination with solvation models SMD or CPCM poorly reproduced the experimental activation-free energies for reactions between eight investigated chemical carcinogens and guanine, with Δ(ΔG) differences ranging from 2.80 kcal/mol in the case of BPL (M06-2X/SMD) to 6.88 kcal/mol in the case of EO (M06-2X/CPCM), while VCE posed the only exception with Δ(ΔG) below 1 kcal/mol for combination with either solvation model. From Figure 4, it is also evident that the activation-free energies calculated using M11-L and MN12-L DFT functionals in conjunction with the 6-311++G(d,p) basis set and SMD solvation model provide the best overall agreement with the experimental data for all nine investigated chemical carcinogens. The obtained Δ(ΔG) values range from 0.31 kcal/mol (BPL) to 1.89 kcal/mol (AFB1) in the case of M11-L/SMD and from 0.05 kcal/mol (PO) to −1.86 kcal/mol (CEO) in the case of MN12-L/SMD.

In Table 1, the calculated activation-free energies with M11-L/SMD and MN12-L/SMD methods, the lowest vibrational frequencies and distances between the reactive centers corresponding to reactant and transition states, as well as absolute and relative errors, together with the corresponding experimental activation-free energies for reactions of guanine with nine investigated chemical carcinogens, are collected. The calculated activation-free energies with the corresponding absolute and relative errors as well as vibrational frequencies of reactants and transition states for all tested DFT functionals are provided in Supplementary Material Tables S1–S9.

From Table 1, it can be observed that the lowest obtained real frequencies of optimized reactant structures of guanine and nine investigated chemical carcinogens were between 8.28 cm^−1^ and 45.33 cm^−1^. The obtained single imaginary frequencies of transition states ranging from 207.05 i cm^−1^ to 654.07 i cm^−1^ corresponded to the cleavage of the covalent bond inside the epoxy ring of chemical carcinogen and to the formation of a new covalent bond between the most nucleophilic atom N7 of guanine and the achiral epoxy carbon of the nine chemical carcinogens (Table 1). The *S_N_2* substitution mechanisms for the formation of the nine chemical carcinogen–guanine adducts were proposed (Appendix A). The distances between the reactive centers of the reactants d^R^ lied between 2.79 Å and 4.24 Å and were approximately 1 Å to 2 Å shorter than the corresponding distances in transition states ranging from 1.94 Å to 2.19 Å. The shorter distances correspond to stronger intermolecular interactions in the transition states than in the reactant states. Similar distances between reactive centers in transition state structures d^TS^ obtained with the M11-L/SMD and MN12-L/SMD methods also indicate similar geometries of optimized transition state structures. The predicted activation-free energies by applying the M11-L and MN12-L DFT functionals in combination with the flexible 6-311++G(d,p) basis set and SMD solvation model were in the range of 16.99 kcal/mol to 27.16 kcal/mol and from 16.88 kcal/mol to 26.49 kcal/mol, respectively. Among all chemical carcinogens, the $\Delta G_{smd}^{‡}$ obtained with the M11-L/SMD and MN12-L/SMD methods was the lowest for the reaction of AFB1 with guanine (16.99 kcal/mol and 16.88 kcal/mol, respectively), indicating that the reaction of guanine with AFB1 is the fastest. The $\Delta G_{smd}^{‡}$ obtained with M11-L/SMD and MN12-L/SMD is the highest for the reaction of guanine with styrene oxide (27.16 kcal/mol and 25.50 kcal/mol, respectively), indicating the slowest reaction of guanine with this chemical carcinogen. From Table 1, it is also evident that the DFT functionals M11-L/SMD and MN12-L/SMD predicted very similar values of activation-free energies for reactions of all nine investigated chemical carcinogens with guanine (difference ~1 kcal/mol).

The largest discrepancy between the calculated and experimental activation-free energy was found for the reaction of AFB1 exo-8.9-epoxide and of vinyl carbamate epoxide with guanine in the case of M11-L/SMD, as well as for the reaction of chloroethylene oxide and AFB1 exo-8.9-epoxide in the case of MN12-L/SMD. The corresponding absolute errors |Δ(ΔG)| were 1.89 kcal/mol and 1.75 kcal/mol for M11-L/SMD, respectively, as well as 1.86 kcal/mol and 1.78 kcal/mol for MN12-L/SMD, respectively. In addition, the corresponding relative errors obtained with M11-L/SMD for guanine reactions with AFB1 exo-8.9-epoxide and vinyl carbamate were 0.12 and 0.08, respectively, as well as 0.09 and 0.12, respectively, for chloroethylene oxide and AFB1 exo-8.9-epoxide reactions with guanine obtained using MN12-L/SMD. However, the mean absolute error for the test set of nine investigated chemical carcinogens reacting with guanine was found to be 1.08 kcal/mol for M11-L/SMD and 1.11 kcal/mol for MN12-L/SMD, which corresponds to an acceptable computational accuracy of 1 kcal/mol. The mean absolute and relative errors for all tested functionals are provided in Supplementary Material Table S10. As can be observed from Figure 4 and Table S10, the DFT functionals N12SX/SMD, M06HF/SMD, and WB97XD/SMD may also provide reliable kinetic data for some chemical carcinogens (their mean absolute errors were 2.28, 2.39, and 2.41 kcal/mol, respectively); however, their application is not recommended due to the very poor agreement with experimental values in the case of AFB1 exo-8,9-epoxide (Δ(ΔG) values were 6.38, 6.51, and 5.22 kcal/mol, respectively).

**Figure 5:**
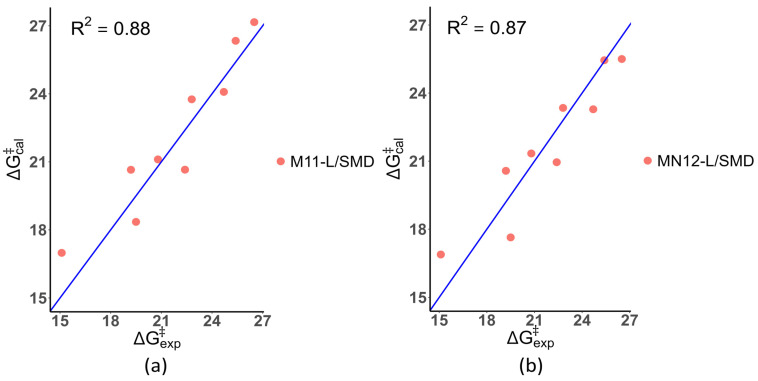
The correlation between the calculated activation-free energies using (**a**) M11-L/SMD and (**b**) MN12-L/SMD methods, respectively, and the experimental activation-free energies for reactions between nine investigated chemical carcinogens and guanine.

The correlation between the calculated activation-free energies using (a) M11-L/SMD and (b) MN12-L/SMD and the experimental activation-free energies for the reactions between the nine investigated chemical carcinogens and guanine is presented in Figure 5. The obtained R^2^ values for M11-L/SMD and MN12-L/SMD were 0.88 and 0.87, respectively, indicating a high correlation between the calculated and experimental activation-free energies for reactions between guanine and the nine investigated chemical carcinogens.

**Figure 6:**
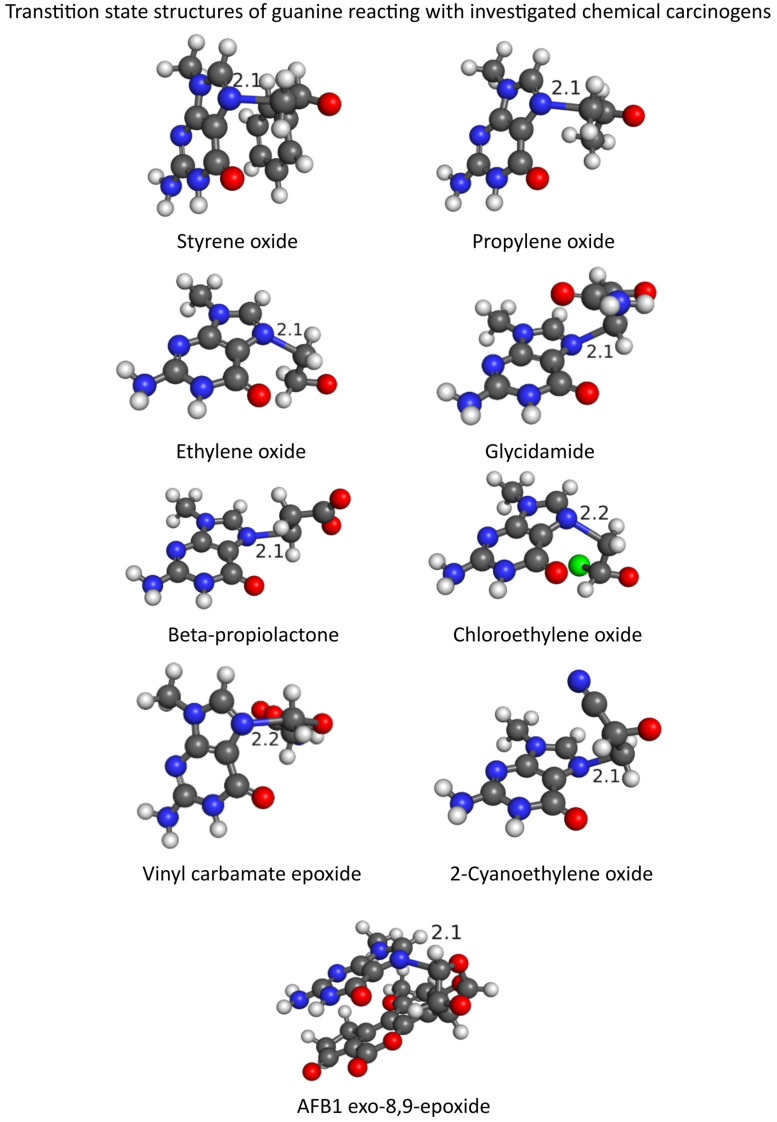
Transition state structures of the nucleophilic attack of the N7 atom of guanine on the achiral carbon in the epoxy ring of the nine investigated chemical carcinogens as predicted by the DFT functional M11-L in combination with the flexible 6-311++G(d,p) basis set and SMD implicit solvation model. The carbon atoms are depicted in gray; the oxygen atoms are red, the nitrogen atoms are blue; the chlorine atom is green; and the hydrogen atoms are white.

The obtained low mean absolute and relative errors as well as high R^2^ values demonstrated the reliability of M11-L and MN12-L DFT functionals in combination with the flexible 6-311++G(d,p) basis set and SMD implicit solvation model to evaluate the reactivity of the nine investigated chemical carcinogens with guanine. The QM-CSA methodology is valid for all chemical carcinogens of the epoxy type and can reliably estimate the CSA. Moreover, the obtained activation-free energies for reactions between nine investigated chemical carcinogens and guanine will allow for the evaluation of the CSA of various natural polyphenolic compounds. The optimized transition state structures obtained with the M11-L/SMD method, corresponding to the studied alkylation reactions between the nine investigated chemical carcinogens and guanine, are presented in Figure 6.

Graphical representations of optimized transition state geometries revealed the formation of a new covalent bond between the N7 atom of guanine and the achiral epoxy carbon atom of the nine investigated chemical carcinogens. The simultaneous cleavage of a covalent bond between the achiral carbon and epoxy oxygen of the chemical carcinogen also confirms the correct transition state structure allocation. The obtained single imaginary frequencies with normal modes following this reaction path for all transition state structures (Table 1) correspond to the proposed *S_N_2* reaction mechanism, which is also in agreement with the previously published scientific literature [2,3,4,5,6,7,8,9,10].

### 3.2. Application of the QM-CSA Protocol: Mechanistic Insights into Alkylation Reactions of Nine Investigated Chemical Carcinogens with EGCG, EGC, and (+)-Catechin

In this subsection, the application of the QM-CSA protocol to the alkylation reactions between the nine investigated chemical carcinogens and three catechins, namely EGCG, EGC, and (+)-catechin, is presented. The previously discussed results revealed that the most accurate $\Delta G_{smd}^{‡}$ for the alkylation reactions of nine investigated chemical carcinogens with guanine can be obtained using the DFT functional M11-L in combination with the flexible 6-311++G(d,p) basis set and implicit solvation model SMD. The mean absolute difference between the experimental and calculated activation-free energies with the M11-L/SMD method for the reactions between the nine chemical carcinogens and guanine was approximately 1 kcal/mol (1.08 kcal/mol), which corresponds to an acceptable computational error. In addition, the MN12-L functional in combination with the flexible 6-311++G(d,p) basis set and SMD solvation model reproduced the experimental values for the reactions between the same nine chemical carcinogens and guanine with an acceptable mean computational error (1.11 kcal/mol) as well. Therefore, the DFT functionals M11-L/SMD and MN12-L/SMD were also applied to calculate the corresponding $\Delta G_{smd}^{‡}$ for the reactions of nine investigated chemical carcinogens with EGCG, EGC, and (+)-catechin, as well as to calculate $\Delta G_{smd}^{‡}$ for the reactions of the same nine chemical carcinogens with a reference compound glutathione. $\Delta G_{smd}^{‡}$ values calculated with the M11-L/SMD and MN12-L/SMD methods are also expected to be in very good agreement with the experimental $\Delta G_{exp}^{‡}$ values for the reactions of the same nine chemical carcinogens with EGCG, EGC, (+)-catechin, and glutathione. The calculated $\Delta G_{smd}^{‡}$ values for the reactions of EGCG, EGC, and (+)-catechin with the nine chemical carcinogens were then compared to the calculated $\Delta G_{smd}^{‡}$ values for the reactions of the same chemical carcinogens with guanine and glutathione. A lower $\Delta G_{smd}^{‡}$ indicates a faster reaction of the polyphenolic compound with the chemical carcinogen. We hypothesized that the $\Delta G_{smd}^{‡}$ of the reaction between the nine chemical carcinogens and EGCG, EGC, or (+)-catechin is lower than the $\Delta G_{smd}^{‡}$ of the competing reaction between the same chemical carcinogens and guanine (Figure 3 in the Section 2).

In Table 2, the calculated $\Delta G_{smd}^{‡}$ with the M11-L/SMD method for alkylation reactions between the nine investigated chemical carcinogens and EGCG, EGC, (+)-catechin as well as glutathione, the lowest vibrational frequencies of reactant states, imaginary frequencies of transition states, the corresponding distances between the reactive centers in reactants and transition states, and relative CSA values $r^{glutathione}$ are collected. The calculated $\Delta G_{guanine}^{‡}$ values for the reactions between the nine chemical carcinogens and guanine are also provided. The corresponding values obtained with the MN12-L functional in combination with the flexible 6-311++G(d,p) basis set and SMD implicit solvation model are reported in Table S11 of the Supplementary Materials.

As can be observed from Table 2, the correctly optimized structures of the reactant state must exhibit only real vibrational frequencies. The lowest obtained real vibrational frequencies of reactants EGCG, EGC, (+)-catechin, and glutathione with nine investigated chemical carcinogens were between 3.85 cm^−1^ and 27.95 cm^−1^. The correctly optimized transition state structure is characterized by exactly one imaginary vibrational frequency, which in normal mode must represent the reaction coordinate, corresponding to the cleavage of the covalent bond inside the epoxy ring of the investigated chemical carcinogen and the formation of a new covalent bond between the nonchiral carbon atom of the chemical carcinogen and the most nucleophilic oxygen atom of EGCG, EGC, and (+)-catechin or the sulfur atom on glutathione. The obtained single imaginary frequencies ranging from 143.49 i cm^−1^ to 712.15 i cm^−1^ indeed corresponded to the formation of the new covalent bond between the most nucleophilic oxygen of EGCG, EGC, and (+)-catechin or the sulfur on glutathione and the achiral epoxy carbon of all nine investigated chemical carcinogens (Table 2).

Much weaker intermolecular interactions in the reactant than in the transition states are reflected in larger values of d^R^ compared to d^TS^, indicating a much shallower potential hypersurface in the case of reactants. The distances between the reactive centers of the reactants lie between 3.06 Å and 4.82 Å and are approximately 1 Å to 2 Å shorter than in the corresponding transition states, with distances ranging from 1.99 Å to 3.06 Å. From Table 2, it can also be observed that the distances between reactive centers in transition states were between 0.5 Å and 0.7 Å shorter for the reactions of all investigated chemical carcinogens with the three studied catechins than in the case of glutathione due to a larger van der Waals radius of the nucleophilic sulfur.

The predicted activation-free energies using the M11-L/SMD method were in the range of 15.70 kcal/mol to 25.71 kcal/mol, 11.64 kcal/mol to 22.12 kcal/mol, and 8.02 kcal/mol to 20.32 kcal/mol for the reactions of chemical carcinogens with EGCG, EGC, and (+)-catechin, respectively. Among all investigated chemical carcinogens, the $\Delta G_{smd}^{‡}$ obtained with the M11-L/SMD method is the lowest for the reaction of chloroethylene oxide with (+)-catechin (8.02 kcal/mol). Therefore, the reaction of chloroethylene oxide with (+)-catechin is the fastest. The $\Delta G_{smd}^{‡}$ obtained with the M11-L/SMD is the highest for the reaction of EGCG with propylene oxide (25.71 kcal/mol). Therefore, this reaction is the slowest.

Moreover, quantum mechanical calculations with the M11-L/SMD method predicted significantly lower activation-free energies (by 2 to 10 kcal/mol) for reactions of all investigated chemical carcinogens with (+)-catechin and EGC than for the competing reactions with the most reactive DNA base, guanine. EGCG demonstrated lower activation-free energies (by >1 kcal/mol) for reactions with six investigated chemical carcinogens than guanine and similar activation-free energies for reactions with propylene oxide, ethylene oxide, and AFB1 exo-8.9-epoxide (difference <1 kcal/mol).

The calculated ratios $r^{glutathione}$ were higher than one for reactions of all nine investigated chemical carcinogens with EGCG, six investigated chemical carcinogens with EGC, and five investigated chemical carcinogens with (+)-catechin, indicating a higher reactivity of glutathione. In the case of reactions of EGC with glycidamide, vinyl carbamate epoxide, and beta-propiolactone as well as of (+)-catechin with glycidamide, beta-propiolactone, chloroethylene oxide, and 2-cyanoethylene oxide, the calculated ratios $r^{glutathione}$ were lower than one, indicating a higher relative scavenging potential of EGC and (+)-catechin towards these chemical carcinogens than glutathione.

**Figure 7:**
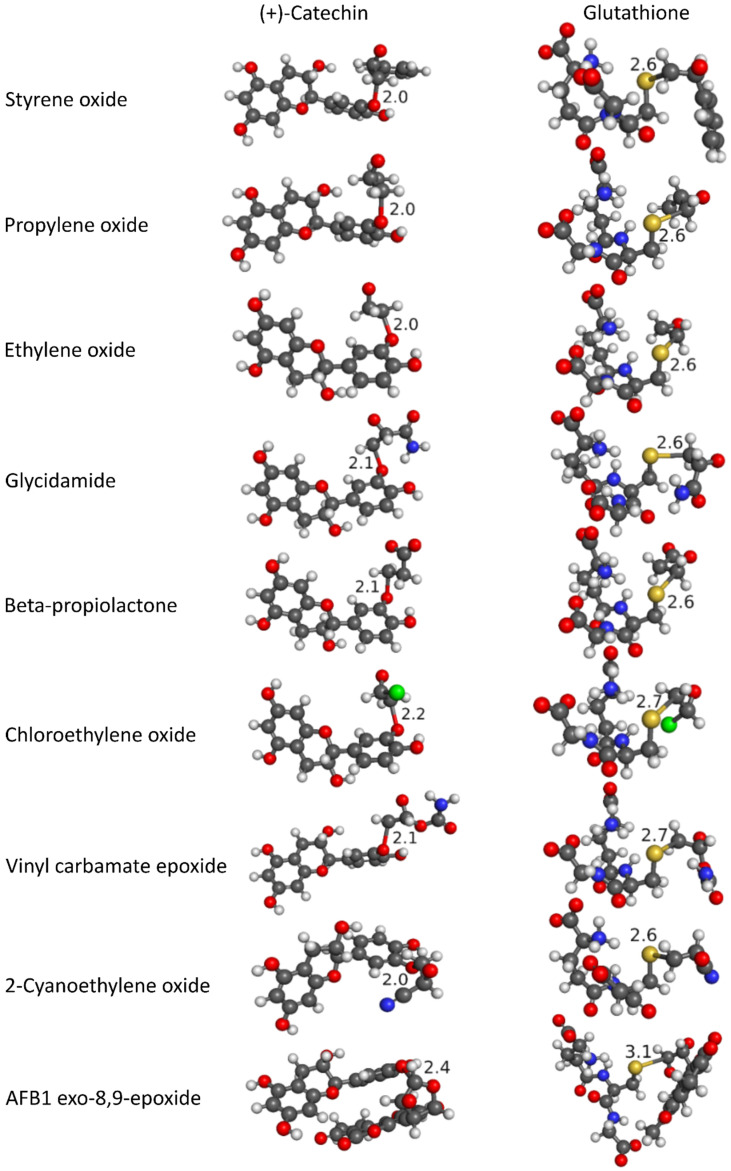
Transition state structures of alkylation reactions of the most efficient polyphenolic scavenger (+)-catechin and the reference compound glutathione with investigated chemical carcinogens obtained using the DFT functional M11-L in combination with the flexible 6-311++G(d,p) basis set and SMD implicit solvation model. The carbon atoms are gray; the oxygen atoms are red; the nitrogen atoms are blue; the sulfur atom is yellow; the chlorine atom is green; and the hydrogen atoms are white.

From the results collected in Table 2, the following general order of reactivity with the nine investigated chemical carcinogens can be established: (+)-catechin > EGC > EGCG. Nonepistructured (+)-catechin, therefore, represents the most efficient scavenger of investigated chemical carcinogens, although epistructured EGC and EGCG also represent potential carcinogen scavengers. The observed order of reactivity may in part be a consequence of the least sterically hindered structure of nonepistructured (+)-catechin, which allowed for the closest contact with investigated chemical carcinogens, whereas the reactions of chemical carcinogens with larger epistructured catechins EGC and EGCG were more sterically hindered. Graphical representations of transition state structures for the reactions of the most efficient polyphenolic scavenger (+)-catechin and the reference compound glutathione with investigated chemical carcinogens are depicted in Figure 7.

From Figure 7, the formation of a new covalent bond between the most nucleophilic phenolic oxygen atom of (+)-catechin or the sulfur atom of glutathione and the achiral epoxy carbon of nine investigated chemical carcinogens can be observed. The correct transition state structure allocation was also confirmed by the simultaneous cleavage of the covalent bond connecting this achiral carbon with the epoxy oxygen of the chemical carcinogen. The exclusively obtained single imaginary vibrational frequencies (Table 2) corresponding to this reaction path additionally confirm the validity of the proposed *S_N_2* reaction mechanism for alkylation reactions of polyphenolic compounds with investigated chemical carcinogens [43,44].

Based on the obtained results, we can safely assume that the reactions between a large majority of investigated chemical carcinogens and (+)-catechin, EGC, as well as EGCG, would occur faster than the competing reactions between guanine and the same chemical carcinogens, meaning that the studied catechins could successfully prevent chemical carcinogen-induced DNA adduct formation. The obtained results also revealed a novel possible mechanism of the chemoprotective effects of EGCG, EGC, and (+)-catechin against nine investigated chemical carcinogens, potentially contributing to the prevention of chemical carcinogen–DNA adduct formation.

## 4. Conclusions

In this study, a novel quantum mechanics-based QM-CSA protocol to evaluate and compare the potential of polyphenolic compounds as scavengers of chemical carcinogens of the epoxy type under physiological conditions is developed. The QM-CSA protocol is based on kinetic calculations and represents a unique and quantitative approach to predicting the carcinogen-scavenging activity (CSA) of polyphenolic compounds by applying two scales: the absolute scale based on the comparison of activation-free energies of a series of polyphenols with guanine and a relative scale based on the comparison with glutathione as a reference compound. The reliability of QM-CSA was demonstrated by comparison of the calculated activation-free energies with the corresponding experimental activation-free energies for alkylation reactions between nine investigated chemical carcinogens and the most reactive DNA base, guanine.

The importance of the selection of an appropriate combination of DFT functionals, namely M11-L or MN12-L, flexible basis set 6-311++G(d,p), and implicit solvation model SMD to achieve the best agreement with experimental data under physiological conditions was demonstrated. The functionals M11-L and MN12-L, in conjunction with the SMD solvation model, were identified as the most reliable DFT methods to evaluate the reactivity of nine investigated chemical carcinogens of the epoxy type with guanine among the 14 tested functionals. The uncertainties of the proposed QM-CSA methodology have been proven to be similar to experimental ones with mean absolute errors of 1.08 kcal/mol and 1.11 kcal/mol for M11-L/SMD and MN12-L/SMD, respectively.

Moreover, the QM-CSA protocol was also applied to evaluate the CSA of a series of three catechins, namely EGCG, EGC, and (+)-catechin, and to compare their potential as scavengers of nine investigated chemical carcinogens at physiological conditions with guanine and glutathione. The following order of reactivity in the series of three catechins was determined: (+)-catechin > EGC > EGCG, indicating that (+)-catechin represents the most efficient scavenger of the investigated chemical carcinogens in comparison with guanine and glutathione. The proposed *S_N_2* reaction mechanism was also confirmed.

To conclude, the QM-CSA protocol allows for the estimation of activation-free energies, the identification of polyphenolic compounds with the highest carcinogen scavenging potential, the prediction of order/trend of scavenging activity, and insights into the molecular mechanisms of the studied alkylation reactions. The applications of the QM-CSA protocol are intended to increase the kinetic data on alkylation reactions between polyphenolic compounds and chemical carcinogens of the epoxy type, which are currently lacking in the scientific literature, and to facilitate the design of efficient novel pharmacological strategies for cancer prevention.

## Figures and Tables

**Table 1:** Activation-free energies, lowest vibrational frequencies, and distances between the reactive centers of reactant and transition states, as well as absolute and relative errors together with corresponding experimental activation-free energies for the reactions between nine studied chemical carcinogens and guanine at the M11-L/SMD and MN12-L/SMD levels of theory.

Functional/Solvation Model	$\Delta G_{SMD}^{‡}$ (kcal/mol) ^a^	ω^TS^ (i cm^−1^) ^b^	ω^R^ (cm^−1^) ^c^	d^TS^ (Å) ^d^	d^R^ (Å) ^e^	|Δ(ΔG)| (kcal/mol) ^f^	$\frac{\left|\Delta \left(\Delta G\right)\right|}{\Delta G_{exp}^{‡}}$ (/) ^g^	$\Delta G_{exp}^{‡}$ (kcal/mol) ^h^
**Styrene Oxide**								
**M11-L/SMD**	27.16	640.08	17.63	1.94	3.34	0.66	0.02	26.5
**MN12-L/SMD**	25.50	577.35	22.26	1.94	3.13	1.00	0.04	26.5
**Propylene Oxide**								
**M11-L/SMD**	26.34	629.23	29.99	2.06	3.82	0.94	0.04	25.4
**MN12-L/SMD**	25.45	569.04	30.07	2.05	3.17	0.05	0.002	25.4
**Ethylene Oxide**								
**M11-L/SMD**	24.08	613.17	11.77	2.07	2.79	0.62	0.02	24.7
**MN12-L/SMD**	23.29	580.76	8.28	2.06	3.24	1.41	0.05	24.7
**Glycidamide**								
**M11-L/SMD**	23.76	654.07	24.06	2.08	3.70	0.96	0.04	22.8
**MN12-L/SMD**	23.35	598.03	24.22	2.06	3.26	0.55	0.02	22.8
**Vinyl Carbamate Epoxide**								
**M11-L/SMD**	20.65	543.75	29.15	2.15	3.49	1.75	0.08	22.4
**MN12-L/SMD**	20.95	515.44	25.84	2.13	3.36	1.45	0.06	22.4
**Beta-propiolactone**								
**M11-L/SMD**	21.11	583.95	16.85	2.13	3.57	0.31	0.01	20.8
**MN12-L/SMD**	21.34	562.69	17.17	2.13	3.39	0.54	0.03	20.8
**Chloroethylene Oxide**								
**M11-L/SMD**	18.35	539.26	23.65	2.19	3.59	1.15	0.06	19.5
**MN12-L/SMD**	17.64	515.29	36.83	2.16	3.19	1.86	0.09	19.5
**2-Cyanoethylene Oxide**								
**M11-L/SMD**	20.65	646.51	20.00	2.10	3.32	1.45	0.08	19.2
**MN12-L/SMD**	20.57	616.92	45.33	2.08	3.50	1.37	0.07	19.2
**AFB1 Exo-8.9-Epoxide**								
**M11-L/SMD**	16.99	225.87	21.53	2.12	4.21	1.89	0.12	15.1
**MN12-L/SMD**	16.88	207.05	26.03	2.12	4.14	1.78	0.12	15.1

^a^ Activation-free energy is calculated with the M11-L or MN12-L functionals in combination with the flexible 6-311++G(d,p) basis set and SMD solvation model. ^b^ The exact imaginary vibrational frequency of the transition state structure. ^c^ The lowest real vibrational frequency of the reactant state structure. ^d^ The distance in the transition state structure between the most nucleophilic nitrogen atom N7 on guanine and the achiral electrophilic epoxy carbon of the chemical carcinogen. ^e^ The distance in the reactant structure between the most nucleophilic atom N7 of guanine and the chiral electrophilic epoxy carbon of chemical carcinogen. ^f^ The absolute error between the calculated and experimental activation-free energies for reactions between investigated chemical carcinogens and guanine. ^g^ The relative error between the calculated and experimental activation-free energies for reactions between investigated chemical carcinogens and guanine. ^h^ Experimental activation-free energies for reactions of investigated chemical carcinogens with guanine.

**Table 2:** Computational results obtained with the M11-L functional in combination with the flexible 6-311++G(d,p) basis set and SMD implicit solvation model for the alkylation reactions of the nine investigated chemical carcinogens with EGCG, EGC, (+)-catechin, and glutathione.

Functional/Solvation Model M11-L/SMD	$\Delta G_{SMD}^{‡}$ (kcal/mol) ^a^	ω^TS^ (i cm^−1^) ^b^	ω^R^ (cm^−1^) ^c^	*d*^TS^ (Å) ^d^	*d*^R^ (Å) ^e^	r (/) ^f^	$\Delta G_{guanine}^{‡}$ (kcal/mol) ^g^
**Styrene Oxide**							
**EGCG**	25.65	585.16	10.48	1.99	3.35	1.53	27.16
**EGC**	19.80	577.89	6.19	2.04	3.67	1.18	27.16
**(+)-Catechin**	20.32	591.12	11.05	2.03	3.14	1.21	27.16
**Glutathione**	16.81	677.09	15.61	2.60	4.14	1.00	27.16
**Propylene Oxide**							
**EGCG**	25.71	566.03	14.46	2.00	3.25	1.52	26.34
**EGC**	20.73	581.61	25.13	2.03	3.41	1.23	26.34
**(+)-Catechin**	18.85	555.36	16.31	2.02	3.35	1.12	26.34
**Glutathione**	16.87	663.75	22.00	2.58	3.47	1.00	26.34
**Ethylene Oxide**							
**EGCG**	24.27	566.56	14.55	2.01	3.73	1.70	24.08
**EGC**	22.12	586.59	27.95	2.05	3.85	1.55	24.08
**(+)-Catechin**	17.37	574.66	21.41	2.03	3.36	1.22	24.08
**Glutathione**	14.25	675.23	27.41	2.60	3.69	1.00	24.08
**Glycidamide**							
**EGCG**	22.07	552.28	16.34	2.02	4.48	1.09	23.76
**EGC**	19.35	562.81	8.97	2.06	4.64	0.95	23.76
**(+)-Catechin**	19.46	583.34	11.72	2.05	3.48	0.96	23.76
**Glutathione**	20.30	694.05	9.16	2.57	4.13	1.00	23.76
**Vinyl Carbamate Epoxide**							
**EGCG**	17.64	564.04	10.75	2.10	3.58	1.47	20.65
**EGC**	11.64	591.42	11.13	2.13	3.54	0.97	20.65
**(+)-Catechin**	13.61	590.77	21.98	2.12	3.70	1.14	20.65
**Glutathione**	11.97	655.05	14.84	2.65	4.82	1.00	20.65
**Beta-propiolactone**							
**EGCG**	16.97	581.69	12.28	2.09	3.06	1.20	21.11
**EGC**	12.85	615.41	17.64	2.10	3.23	0.91	21.11
**(+)-Catechin**	10.65	630.25	16.37	2.08	3.24	0.75	21.11
**Glutathione**	14.19	653.94	12.49	2.62	3.65	1.00	21.11
**Chloroethylene Oxide**							
**EGCG**	15.70	580.33	15.14	2.14	3.82	1.68	18.35
**EGC**	12.86	607.74	17.61	2.16	3.52	1.37	18.35
**(+)-Catechin**	8.02	591.69	9.26	2.15	3.47	0.86	18.35
**Glutathione**	9.36	633.79	23.68	2.74	3.69	1.00	18.35
**2-Cyanoethylene Oxide**							
**EGCG**	20.01	612.51	6.30	2.04	3.30	1.43	20.65
**EGC**	17.97	634.52	5.88	2.07	3.37	1.29	20.65
**(+)-Catechin**	13.95	644.96	3.85	2.05	3.45	0.99	20.65
**Glutathione**	13.99	712.15	9.04	2.63	3.68	1.00	20.65
**AFB1 Exo-8.9-Epoxide**							
**EGCG**	17.15	156.05	14.12	2.40	3.81	1.33	16.99
**EGC**	13.15	204.14	10.80	2.68	3.40	1.02	16.99
**(+)-Catechin**	14.37	143.49	10.84	2.39	3.32	1.11	16.99
**Glutathione**	12.92	245.54	8.88	3.06	3.77	1.00	16.99

^a^ Activation-free energies obtained with the DFT functional M11-L in combination with the flexible 6-311++G(d,p) basis set and SMD implicit solvation model. ^b^ The exact imaginary vibrational frequency of the transition state structure. ^c^ The lowest vibrational frequency of the reactant state structure. ^d^ The distance in the transition state structure between the most nucleophilic phenolic oxygen of EGCG, EGC, (+)-catechin, or the sulfur atom of glutathione, and the achiral electrophilic epoxy carbon of the chemical carcinogen. ^e^ The distance in the reactant structure between the most nucleophilic phenolic oxygen of EGCG, EGC, (+)-catechin, or the sulfur atom of glutathione, and the achiral electrophilic epoxy carbon of the chemical carcinogen. ^f^ The ratio of activation-free energies between the investigated polyphenols, namely EGCG, EGC, (+)-catechin, and glutathione, allowing for the determination of the relative CSA. ^g^ Activation-free energy for the reaction with guanine was calculated using the DFT functional M11-L in combination with the flexible 6-311++G(d,p) basis set and SMD implicit solvation model.

## Data Availability

The original contributions presented in the study are included in the article and Supplementary Material, further inquiries can be directed to the corresponding authors.

## Supplementary Materials

The following supporting information can be downloaded at: https://www.mdpi.com/article/10.3390/foods13172708/s1, Tables S1–S9. The activation-free energies for the reaction between nine investigated chemical carcinogens and guanine calculated with 14 DFT functionals in conjunction with the 6-311++G(d,p) flexible basis set and the SMD or CPCM solvation model, together with the corresponding absolute and relative errors, as well as the frequencies corresponding to reactant and transition state structures; Table S10. The average absolute and relative errors for the reactions between nine investigated chemical carcinogens and guanine calculated with 14 tested DFT functionals in conjunction with the SMD and CPCM solvation models; Table S11. Computational results obtained with the MN12-L functional in conjunction with the 6-311++G(d,p) flexible basis set and SMD implicit solvation model for the reactions of nine investigated chemical carcinogens with EGCG, EGC, (+)-catechin, and glutathione.

## Appendix A

The proposed *S_N_2* mechanisms for the alkylation reactions of nine investigated chemical carcinogens with (**a**) guanine and (**b**) glutathione are depicted in Scheme A1, Scheme A2, Scheme A3, Scheme A4, Scheme A5, Scheme A6, Scheme A7, Scheme A8 and Scheme A9.
