# Supplementary material for: QM-CSA: A Novel Quantum Mechanics-Based Protocol for Evaluation of the Carcinogen-Scavenging Activity of Polyphenolic Compounds
Source: Foods. 2024 Aug 27;13(17):2708. doi: 10.3390/foods13172708 (PMC11394233; doi:10.3390/foods13172708)
Supplement: Supplementary file 1 [file foods-13-02708-s001.zip › foods-3149711-supplementary.pdf]

Supplementary Materials

QM-CSA: A Novel Quantum Mechanics-Based Protocol for Evaluation of the Carcinogen-Scavenging Activity of Polyphenolic Compounds

Veronika Furlan [1,2], Jelena Tošović [1,*] and Urban Bren [1,2,3,*]

[1] Faculty of Chemistry and Chemical Engineering, University of Maribor, Smetanova 17,

SI-2000 Maribor, Slovenia; veronika.furlan@um.si

[2] Institute of Environmental Protection and Sensors, Beloruska Ulica 7, SI-2000 Maribor, Slovenia

[3] Faculty of Mathematics, Natural Sciences and Information Technologies, University of Primorska,

Glagoljaška 8, SI-6000 Koper, Slovenia

* Correspondence: jelena.tosovic@guest.um.si (J.T.); urban.bren@um.si (U.B.)

Table S1. The activation-free energies for the reaction between styrene oxide and guanine calculated with 14 DFT functionals in conjunction with 6-311++G(d,p) flexible basis set and SMD or CPCM solvation model together with the corresponding absolute and relative errors, as well as obtained frequencies corresponding to reactant and transition state structures.

Functional	Solvation model	$\Delta G^{\ddagger}$ [kcal/mol][a]	$\lvert\Delta(\Delta G)\rvert$ [kcal/mol][b]	$\frac{\lvert\Delta(\Delta G)\rvert}{\Delta G^{\ddagger}_{exp}}$ [/][c]	ω^{TS} [i cm^{-1}][d]	ω^{R} [cm^{-1}][e]
**Styrene oxide**						
B3LYP-D3	SMD	24.57	1.93	0.07	563.45	15.21
B3LYP-D3	CPCM	23.06	3.44	0.13	492.51	24.52
M05-2X	SMD	28.09	1.59	0.06	682.54	1.43
M05-2X	CPCM	30.52	4.02	0.15	651.68	21.27
M06-2X	SMD	29.86	3.36	0.13	677.92	17.88
M06-2X	CPCM	33.34	6.84	0.26	674.06	14.74
M06-HF	SMD	26.60	0.10	0.00	688.63	15.93
M06-HF	CPCM	29.89	3.39	0.13	720.30	28.15
M08-HX	SMD	26.64	0.14	0.01	733.55	18.81
M08-HX	CPCM	28.26	1.76	0.07	708.81	27.32
M11	SMD	29.48	2.98	0.11	727.56	19.58
M11	CPCM	32.45	5.95	0.22	697.75	21.99
M11-L	SMD	27.16	0.66	0.02	640.08	17.63
M11-L	CPCM	26.69	0.19	0.01	557.03	10.72
MN12-L	SMD	25.50	1.00	0.04	577.35	22.26
MN12-L	CPCM	23.70	2.80	0.11	533.54	22.57
MN12-SX	SMD	25.02	1.48	0.06	621.34	20.94
MN12-SX	CPCM	30.07	3.57	0.13	588.98	24.59
MN15	SMD	27.96	1.46	0.06	624.78	20.68
MN15	CPCM	28.17	1.67	0.06	604.88	27.17
MN15-L	SMD	22.58	3.92	0.15	557.16	24.34
MN15-L	CPCM	25.72	0.78	0.03	522.71	27.88
N12	SMD	26.71	0.21	0.01	531.38	3.00
N12	CPCM	29.47	2.97	0.11	456.54	0.79
N12-SX	SMD	26.73	0.23	0.01	601.63	4.34
N12-SX	CPCM	27.93	1.43	0.05	553.33	4.50
Wb97XD	SMD	27.93	1.43	0.05	630.69	2.90
Wb97XD	CPCM	29.39	2.89	0.11	600.01	20.16

[a] Activation-free energies for reactions of styrene oxide with guanine calculated with 14 DFT functionals in conjunction with SMD and CPCM solvation models. [b] The absolute error between calculated and experimental activation-free energies for reactions between styrene oxide and guanine. [c] The relative error between calculated and experimental activation-free energies for reactions between styrene oxide and guanine. [d] The exactly one imaginary vibrational frequency of the transition state structure. [e] The lowest vibrational frequency of the reactant state structure.

Table S2. The activation-free energies for the reaction between propylene oxide and guanine calculated with 14 DFT functionals in conjunction with 6-311++G(d,p) flexible basis set and SMD or CPCM solvation model together with the corresponding absolute and relative errors, as well as obtained frequencies corresponding to reactant and transition state structures.

Functional	Solvation model	$\Delta G^{\ddagger}$ [kcal/mol][a]	$\lvert\Delta(\Delta G)\rvert$ [kcal/mol][b]	$\frac{\lvert\Delta(\Delta G)\rvert}{\Delta G^{\ddagger}_{exp}}$ [/][c]	ω^{TS} [i cm^{-1}][d]	ω^{R} [cm^{-1}][e]
**Propylene oxide**						
B3LYP-D3	SMD	23.78	1.62	0.06	551.19	21.25
B3LYP-D3	CPCM	24.01	1.39	0.05	474.79	8.35
M05-2X	SMD	28.41	3.01	0.12	672.50	20.72
M05-2X	CPCM	29.84	4.44	0.17	642.43	15.06
M06-2X	SMD	30.75	5.35	0.21	665.36	18.95
M06-2X	CPCM	32.25	6.85	0.27	662.55	21.57
M06-HF	SMD	28.86	3.46	0.14	673.02	9.11
M06-HF	CPCM	32.03	6.63	0.26	700.94	19.60
M08-HX	SMD	29.65	4.25	0.17	732.18	23.27
M08-HX	CPCM	31.23	5.83	0.23	709.80	13.35
M11	SMD	30.78	5.38	0.21	719.06	31.98
M11	CPCM	31.54	6.14	0.24	690.24	27.06
M11-L	SMD	26.34	0.94	0.04	629.23	29.99
M11-L	CPCM	28.39	2.99	0.12	546.26	17.52
MN12-L	SMD	25.45	0.05	0.00	569.04	30.07
MN12-L	CPCM	26.77	1.37	0.05	523.80	27.73
MN12-SX	SMD	27.33	1.93	0.08	610.78	30.57
MN12-SX	CPCM	30.06	4.66	0.18	577.61	27.44
MN15	SMD	28.71	3.31	0.13	615.48	19.16
MN15	CPCM	29.40	4.00	0.16	591.93	13.69
MN15-L	SMD	28.71	3.31	0.13	552.59	20.55
MN15-L	CPCM	31.51	6.11	0.24	516.48	22.27
N12	SMD	25.74	0.34	0.01	522.57	3.77
N12	CPCM	28.80	3.40	0.13	450.70	2.84
N12-SX	SMD	26.53	1.13	0.04	587.67	17.57
N12-SX	CPCM	29.73	4.33	0.17	546.03	3.97
Wb97XD	SMD	28.71	3.31	0.13	620.41	6.11
Wb97XD	CPCM	27.84	2.44	0.10	584.26	29.19

[a] Activation-free energies for reactions of propylene oxide with guanine calculated with 14 DFT functionals in conjunction with SMD and CPCM solvation models. [b] The absolute error between calculated and experimental activation-free energies for reactions between propylene oxide and guanine. [c] The relative error between calculated and experimental activation-free energies for reactions between propylene oxide and guanine. [d] The exactly one imaginary vibrational frequency of the transition state structure. [e] The lowest vibrational frequency of the reactant state structure.

Table S3. The activation-free energies for the reaction between ethylene oxide and guanine calculated with 14 DFT functionals in conjunction with 6-311++G(d,p) flexible basis set and SMD or CPCM solvation model together with the corresponding absolute and relative errors, as well as obtained frequencies corresponding to reactant and transition state structures.

Functional	Solvation model	$\Delta G^{\ddagger}$ [kcal/mol][a]	$\lvert\Delta(\Delta G)\rvert$ [kcal/mol][b]	$\frac{\lvert\Delta(\Delta G)\rvert}{\Delta G^{\ddagger}_{exp}}$ [/][c]	ω^{TS} [i cm^{-1}][d]	ω^{R} [cm^{-1}][e]
			**Ethylene oxide**			
B3LYP-D3	SMD	20.84	3.86	0.16	516.90	35.00
B3LYP-D3	CPCM	25.57	0.87	0.04	475.33	23.41
M05-2X	SMD	26.48	1.78	0.07	681.18	26.86
M05-2X	CPCM	29.77	5.07	0.21	650.65	14.99
M06-2X	SMD	29.67	4.97	0.20	668.19	5.01
M06-2X	CPCM	31.58	6.88	0.28	667.43	29.41
M06-HF	SMD	26.48	1.78	0.07	673.36	32.63
M06-HF	CPCM	30.57	5.87	0.24	706.50	30.32
M08-HX	SMD	29.15	4.45	0.18	740.27	15.35
M08-HX	CPCM	30.72	6.02	0.24	718.42	31.48
M11	SMD	29.04	4.34	0.18	724.38	13.16
M11	CPCM	29.93	5.23	0.21	695.23	31.61
M11-L	SMD	24.08	0.62	0.02	631.17	11.77
M11-L	CPCM	27.85	3.15	0.13	548.76	29.61
MN12-L	SMD	23.29	1.41	0.05	580.76	8.28
MN12-L	CPCM	28.12	3.42	0.14	520.66	25.84
MN12-SX	SMD	29.10	4.40	0.18	621.87	15.72
MN12-SX	CPCM	29.92	5.22	0.21	580.06	26.98
MN15	SMD	27.45	2.75	0.11	622.72	10.01
MN15	CPCM	30.35	5.65	0.23	596.01	1.53
MN15-L	SMD	27.47	2.77	0.11	567.24	38.29
MN15-L	CPCM	30.34	5.64	0.23	519.95	35.77
N12	SMD	24.88	0.18	0.01	524.16	3.13
N12	CPCM	28.07	3.37	0.14	460.61	3.66
N12-SX	SMD	26.96	2.26	0.09	596.46	5.44
N12-SX	CPCM	28.87	4.17	0.17	550.24	4.73
Wb97XD	SMD	24.92	0.22	0.01	626.58	23.49
Wb97XD	CPCM	30.64	5.94	0.24	581.97	21.79

[a] Activation-free energies for reactions of ethylene oxide with guanine calculated with 14 DFT functionals in conjunction with SMD and CPCM solvation models. [b] The absolute error between calculated and experimental activation-free energies for reactions between ethylene oxide and guanine. [c] The relative error between calculated and experimental activation-free energies for reactions between ethylene oxide and guanine. [d] The exactly one imaginary vibrational frequency of the transition state structure. [e] The lowest vibrational frequency of the reactant state structure.

Table S4. The activation-free energies for the reaction between glycidamide and guanine calculated with 14 DFT functionals in conjunction with 6-311++G(d,p) flexible basis set and SMD or CPCM solvation model together with the corresponding absolute and relative errors, as well as obtained frequencies corresponding to reactant and transition state structures.

Functional	Solvation model	$\Delta G^{\ddagger}$ [kcal/mol][a]	$\lvert\Delta(\Delta G)\rvert$ [kcal/mol][b]	$\frac{\lvert\Delta(\Delta G)\rvert}{\Delta G^{\ddagger}_{exp}}$ [/][c]	ω^{TS} [i cm^{-1}][d]	ω^{R} [cm^{-1}][e]
			**Glycidamide**			
B3LYP-D3	SMD	20.98	1.82	0.08	485.19	30.40
B3LYP-D3	CPCM	22.92	0.12	0.01	462.66	11.88
M05-2X	SMD	24.65	1.85	0.08	683.97	13.21
M05-2X	CPCM	28.48	5.68	0.25	646.20	15.59
M06-2X	SMD	27.67	4.87	0.21	660.21	16.36
M06-2X	CPCM	28.83	6.03	0.26	651.18	22.37
M06-HF	SMD	23.88	1.08	0.05	661.83	18.29
M06-HF	CPCM	26.24	3.44	0.15	676.11	26.50
M08-HX	SMD	27.10	4.30	0.19	733.36	29.74
M08-HX	CPCM	28.87	6.07	0.27	707.54	18.05
M11	SMD	27.64	4.84	0.21	721.45	36.71
M11	CPCM	30.07	7.27	0.32	688.94	28.59
M11-L	SMD	23.76	0.96	0.04	654.07	24.06
M11-L	CPCM	26.67	3.87	0.17	541.26	11.73
MN12-L	SMD	23.35	0.55	0.02	598.03	24.22
MN12-L	CPCM	25.50	2.70	0.12	509.86	25.19
MN12-SX	SMD	25.00	2.20	0.10	633.54	22.60
MN12-SX	CPCM	27.54	4.74	0.21	574.18	15.39
MN15	SMD	25.86	3.06	0.13	640.90	28.79
MN15	CPCM	27.70	4.90	0.21	590.76	33.03
MN15-L	SMD	26.80	4.00	0.18	579.41	40.32
MN15-L	CPCM	28.35	5.55	0.24	506.55	28.22
N12	SMD	21.48	1.32	0.06	513.34	3.31
N12	CPCM	22.35	0.45	0.02	461.21	13.04
N12-SX	SMD	23.88	1.08	0.05	608.84	9.85
N12-SX	CPCM	25.75	2.95	0.13	548.16	14.67
Wb97XD	SMD	24.88	2.08	0.09	639.52	32.92
Wb97XD	CPCM	27.61	4.81	0.21	571.04	21.96

[a] Activation-free energies for reactions of glycidamide with guanine calculated with 14 DFT functionals in conjunction with SMD and CPCM solvation models. [b] The absolute error between calculated and experimental activation-free energies for reactions between glycidamide and guanine. [c]The relative error between calculated and experimental activation-free energies for reactions between glycidamide and guanine. [d] The exactly one imaginary vibrational frequency of the transition state structure. [e] The lowest vibrational frequency of the reactant state structure.

Table S5. The activation-free energies for the reaction between vinyl carbamate epoxide and guanine calculated with 14 DFT functionals in conjunction with 6-311++G(d,p) flexible basis set and SMD or CPCM solvation model together with the corresponding absolute and relative errors, as well as obtained frequencies corresponding to reactant and transition state structures.

Vinyl carbamate epoxide						
Functional	Solvation model	$\Delta G^{\ddagger}$ [kcal/mol][a]	$\lvert\Delta(\Delta G)\rvert$ [kcal/mol][b]	$\frac{\lvert\Delta(\Delta G)\rvert}{\Delta G^{\ddagger}_{exp}}$ [/][c]	ω^{TS} [i cm^{-1}][d]	ω^{R} [cm^{-1}][e]
B3LYP-D3	SMD	16.55	5.85	0.26	499.06	34.09
B3LYP-D3	CPCM	17.09	5.31	0.24	473.54	16.63
M05-2X	SMD	19.94	2.46	0.11	715.23	33.46
M05-2X	CPCM	20.64	1.76	0.08	650.4	16.97
M06-2X	SMD	22.34	0.06	0.00	696.35	22.32
M06-2X	CPCM	23.11	0.71	0.03	649.23	12.24
M06-HF	SMD	22.24	0.16	0.01	664.92	13.85
M06-HF	CPCM	22.39	0.01	0.00	656.18	7.84
M08-HX	SMD	22.24	0.16	0.01	775.98	35.06
M08-HX	CPCM	22.61	0.21	0.01	718.87	23.15
M11	SMD	22.73	0.33	0.01	761.29	32.55
M11	CPCM	22.83	0.43	0.02	690.64	26.48
M11-L	SMD	20.65	1.75	0.08	543.75	29.15
M11-L	CPCM	17.99	4.41	0.20	520.2	11.53
MN12-L	SMD	20.95	1.45	0.06	515.44	25.84
MN12-L	CPCM	17.52	4.88	0.22	498.51	14.50
MN12-SX	SMD	21.52	0.88	0.04	619.29	22.58
MN12-SX	CPCM	20.44	1.96	0.09	573.41	25.97
MN15	SMD	21.87	0.53	0.02	651.86	24.68
MN15	CPCM	21.00	1.40	0.06	583.32	24.99
MN15-L	SMD	22.36	0.04	0.00	518.92	20.38
MN15-L	CPCM	19.42	2.98	0.13	510.82	23.92
N12	SMD	16.28	6.12	0.27	455.91	4.10
N12	CPCM	17.51	4.89	0.22	435.72	3.83
N12-SX	SMD	17.71	4.69	0.21	559.27	5.44
N12-SX	CPCM	18.53	3.87	0.17	542.32	7.35
Wb97XD	SMD	19.77	2.63	0.12	605.76	15.39
Wb97XD	CPCM	19.00	3.40	0.15	572.28	24.50

[a] Activation-free energies for reactions of vinyl carbamate epoxide with guanine calculated with 14 DFT functionals in conjunction with SMD and CPCM solvation models. [b] The absolute error between calculated and experimental activation-free energies for reactions between vinyl carbamate epoxide and guanine. [c] The relative error between calculated and experimental activation-free energies for reactions between vinyl carbamate epoxide and guanine. [d] The exactly one imaginary vibrational frequency of the transition state structure. [e] The lowest vibrational frequency of the reactant state structure.

Table S6. The activation-free energies for the reaction between beta-propiolactone and guanine calculated with 14 DFT functionals in conjunction with 6-311++G(d,p) flexible basis set and SMD or CPCM solvation model together with the corresponding absolute and relative errors, as well as obtained frequencies corresponding to reactant and transition state structures.

**Beta-propiolactone**						
Functional	Solvation model	$\Delta G^{\ddagger}$ [kcal/mol][a]	$\lvert\Delta(\Delta G)\rvert$ [kcal/mol][b]	$\frac{\lvert\Delta(\Delta G)\rvert}{\Delta G^{\ddagger}_{exp}}$ [/][c]	ω^{TS} [i cm^{-1}][d]	ω^{R} [cm^{-1}][e]
B3LYP-D3	SMD	18.11	2.69	0.13	517.32	23.61
B3LYP-D3	CPCM	18.59	2.21	0.11	510.31	22.32
M05-2X	SMD	21.77	0.97	0.05	648.96	10.91
M05-2X	CPCM	20.35	0.45	0.02	641.94	28.16
M06-2X	SMD	23.60	2.80	0.13	685.11	29.90
M06-2X	CPCM	23.65	2.85	0.14	653.83	28.99
M06-HF	SMD	22.35	1.55	0.07	659.30	34.76
M06-HF	CPCM	20.64	0.16	0.01	632.09	14.03
M08-HX	SMD	22.38	1.58	0.08	739.34	13.45
M08-HX	CPCM	22.67	1.87	0.09	716.59	30.12
M11	SMD	22.30	1.50	0.07	688.41	21.87
M11	CPCM	23.06	2.26	0.11	690.13	27.83
M11-L	SMD	21.11	0.31	0.01	583.95	16.85
M11-L	CPCM	23.01	2.21	0.11	595.45	25.53
MN12-L	SMD	21.34	0.54	0.03	562.69	17.17
MN12-L	CPCM	22.54	1.74	0.08	561.35	23.86
MN12-SX	SMD	22.59	1.79	0.09	625.10	18.20
MN12-SX	CPCM	23.58	2.78	0.13	615.71	26.48
MN15	SMD	22.51	1.71	0.08	625.94	16.87
MN15	CPCM	23.11	2.31	0.11	612.30	21.29
MN15-L	SMD	23.38	2.58	0.12	542.60	19.24
MN15-L	CPCM	24.76	3.96	0.19	539.58	30.13
N12	SMD	18.25	2.55	0.12	508.79	4.31
N12	CPCM	19.77	1.03	0.05	504.97	2.43
N12-SX	SMD	20.76	0.04	0.00	588.70	4.62
N12-SX	CPCM	21.04	0.24	0.01	585.59	14.14
Wb97XD	SMD	22.09	1.29	0.06	621.11	22.08
Wb97XD	CPCM	22.69	1.89	0.09	632.60	22.66

[a] Activation-free energies for reactions of beta-propiolactone with guanine calculated with 14 DFT functionals in conjunction with SMD and CPCM solvation models. [b] The absolute error between calculated and experimental activation-free energies for reactions between beta-propiolactone and guanine. [c]The relative error between calculated and experimental activation-free energies for reactions between beta-propiolactone and guanine. [d] The exactly one imaginary vibrational frequency of the transition state structure. [e] The lowest vibrational frequency of the reactant state structure.

Table S7. The activation-free energies for the reaction between chloroethylene oxide and guanine calculated with 14 DFT functionals in conjunction with 6-311++G(d,p) flexible basis set and SMD or CPCM solvation model together with the corresponding absolute and relative errors, as well as obtained frequencies corresponding to reactant and transition state structures.

Functional	Solvation model	$\Delta G^{\ddagger}$ [kcal/mol][a]	$\lvert\Delta(\Delta G)\rvert$ [kcal/mol][b]	$\frac{\lvert\Delta(\Delta G)\rvert}{\Delta G^{\ddagger}_{exp}}$ [/][c]	ω^{TS} [i cm^{-1}][d]	ω^{R} [cm^{-1}][e]
**Chloroethylene oxide**						
B3LYP-D3	SMD	13.89	5.61	0.29	509.02	22.81
B3LYP-D3	CPCM	13.59	5.91	0.30	497.02	21.66
M05-2X	SMD	19.37	0.13	0.01	724.30	28.95
M05-2X	CPCM	20.20	0.70	0.04	671.87	25.07
M06-2X	SMD	22.45	2.95	0.15	714.84	37.17
M06-2X	CPCM	22.95	3.45	0.18	671.51	25.68
M06-HF	SMD	21.24	1.74	0.09	664.70	33.71
M06-HF	CPCM	23.01	3.51	0.18	679.96	28.74
M08-HX	SMD	22.25	2.75	0.14	788.10	35.54
M08-HX	CPCM	23.29	3.79	0.19	742.81	26.95
M11	SMD	22.74	3.24	0.17	769.53	30.15
M11	CPCM	25.10	5.60	0.29	712.17	19.88
M11-L	SMD	18.35	1.15	0.06	539.26	23.65
M11-L	CPCM	17.53	1.97	0.10	546.98	15.26
MN12-L	SMD	17.64	1.86	0.09	515.29	36.83
MN12-L	CPCM	17.32	2.18	0.11	518.85	20.92
MN12-SX	SMD	19.45	0.05	0.00	599.85	36.3
MN12-SX	CPCM	20.33	0.83	0.04	594.49	17.45
MN15	SMD	20.50	1.00	0.05	650.26	24.99
MN15	CPCM	21.47	1.97	0.10	606.17	19.41
MN15-L	SMD	19.98	0.48	0.02	520.80	30.68
MN15-L	CPCM	20.45	0.95	0.05	523.05	26.75
N12	SMD	13.79	5.71	0.29	448.92	8.04
N12	CPCM	14.15	5.35	0.27	439.20	2.87
N12-SX	SMD	17.43	2.07	0.11	564.11	9.61
N12-SX	CPCM	16.73	2.77	0.14	545.40	9.28
Wb97XD	SMD	18.99	0.51	0.03	587.13	14.79
Wb97XD	CPCM	20.42	0.92	0.05	582.51	7.84

[a] Activation-free energies for reactions of chloroethylene oxide with guanine calculated with 14 DFT functionals in conjunction with SMD and CPCM solvation models. [b] The absolute error between calculated and experimental activation-free energies for reactions between chloroethylene oxide and guanine. [c]The relative error between calculated and experimental activation-free energies for reactions between chloroethylene oxide and guanine. [d] The exactly one imaginary vibrational frequency of the transition state structure. [e] The lowest vibrational frequency of the reactant state structure.

Table S8. The activation-free energies for the reaction between 2-cyanoethylene oxide and guanine calculated with 14 DFT functionals in conjunction with 6-311++G(d,p) flexible basis set and SMD or CPCM solvation model together with the corresponding absolute and relative errors, as well as obtained frequencies corresponding to reactant and transition state structures.

Functional	Solvation model	$\Delta G^{\ddagger}$ [kcal/mol][a]	$\lvert\Delta(\Delta G)\rvert$ [kcal/mol][b]	$\frac{\lvert\Delta(\Delta G)\rvert}{\Delta G^{\ddagger}_{exp}}$ [/][c]	ω^{TS} [i cm^{-1}][d]	ω^{R} [cm^{-1}][e]
**2-Cyanoethylene oxide**						
B3LYP-D3	SMD	20.80	1.60	0.08	518.25	7.92
B3LYP-D3	CPCM	20.88	1.68	0.09	495.41	15.15
M05-2X	SMD	24.56	5.36	0.28	698.08	7.86
M05-2X	CPCM	24.59	5.39	0.28	664.68	22.42
M06-2X	SMD	26.13	6.93	0.36	677.42	12.04
M06-2X	CPCM	25.85	6.65	0.35	666.18	30.49
M06-HF	SMD	24.33	5.13	0.27	665.60	28.80
M06-HF	CPCM	24.03	4.83	0.25	684.28	26.32
M08-HX	SMD	25.34	6.14	0.32	739.41	39.28
M08-HX	CPCM	25.82	6.62	0.34	722.00	9.67
M11	SMD	25.12	5.92	0.31	742.10	21.18
M11	CPCM	25.16	5.96	0.31	705.00	23.85
M11-L	SMD	20.65	1.45	0.08	646.51	20.00
M11-L	CPCM	23.12	3.92	0.20	594.65	23.73
MN12-L	SMD	20.57	1.37	0.07	616.92	45.33
MN12-L	CPCM	22.17	2.97	0.15	538.91	15.47
MN12-SX	SMD	25.14	5.94	0.31	658.53	24.21
MN12-SX	CPCM	24.95	5.75	0.30	599.89	21.34
MN15	SMD	26.10	6.90	0.36	657.64	18.57
MN15	CPCM	25.44	6.24	0.33	612.38	12.03
MN15-L	SMD	25.12	5.92	0.31	610.78	37.88
MN15-L	CPCM	25.11	5.91	0.31	538.56	25.77
N12	SMD	22.12	2.92	0.15	501.23	2.25
N12	CPCM	20.79	1.59	0.08	477.88	8.94
N12-SX	SMD	21.87	2.67	0.14	625.48	4.84
N12-SX	CPCM	23.80	4.60	0.24	570.81	7.63
Wb97XD	SMD	24.22	5.02	0.26	666.42	32.37
Wb97XD	CPCM	24.53	5.33	0.28	593.01	6.40

[a] Activation-free energies for reactions of 2-cyanoethylene oxide with guanine calculated with 14 DFT functionals in conjunction with SMD and CPCM solvation models. [b] The absolute error between calculated and experimental activation-free energies for reactions between 2-cyanoethylene oxide and guanine. [c]The relative error between calculated and experimental activation-free energies for reactions between 2-cyanoethylene oxide and guanine. [d] The exactly one imaginary vibrational frequency of the transition state structure. [e] The lowest vibrational frequency of the reactant state structure.

Table S9. The activation-free energies for the reaction between AFB1 exo-8.9-epoxide and guanine calculated with 14 DFT functionals in conjunction with 6-311++G(d,p) flexible basis set and SMD or CPCM solvation model together with the corresponding absolute and relative errors, as well as obtained frequencies corresponding to reactant and transition state structures.

Functional	Solvation model	$\Delta G^{\ddagger}$ [kcal/mol][a]	$\lvert\Delta(\Delta G)\rvert$ [kcal/mol][b]	$\frac{\lvert\Delta(\Delta G)\rvert}{\Delta G^{\ddagger}_{exp}}$ [/][c]	ω^{TS} [i cm^{-1}][d]	ω^{R} [cm^{-1}][e]
**AFB1 exo-8.9-epoxide**						
B3LYP-D3	SMD	20.96	5.86	0.39	153.76	15.16
B3LYP-D3	CPCM	17.67	2.57	0.17	165.57	10.50
M05-2X	SMD	20.44	5.34	0.35	213.75	23.05
M05-2X	CPCM	21.13	6.03	0.40	293.87	6.06
M06-2X	SMD	21.85	6.75	0.45	213.47	14.74
M06-2X	CPCM	22.20	7.10	0.47	237.51	13.70
M06-HF	SMD	21.61	6.51	0.43	510.34	25.99
M06-HF	CPCM	21.30	6.20	0.41	546.74	18.91
M08-HX	SMD	21.35	6.25	0.41	225.38	27.94
M08-HX	CPCM	21.47	6.37	0.42	294.85	9.71
M11	SMD	20.55	5.45	0.36	192.67	19.81
M11	CPCM	21.56	6.46	0.43	304.45	7.33
M11-L	SMD	16.99	1.89	0.12	228.33	22.73
M11-L	CPCM	20.77	5.67	0.38	242.77	10.22
MN12-L	SMD	16.88	1.78	0.12	207.05	26.03
MN12-L	CPCM	17.50	2.40	0.16	256.48	6.58
MN12-SX	SMD	21.63	6.53	0.43	218.42	22.58
MN12-SX	CPCM	22.01	6.91	0.46	247.21	10.23
MN15	SMD	22.08	6.98	0.46	217.60	15.75
MN15	CPCM	21.96	6.86	0.45	278.43	10.11
MN15-L	SMD	18.03	2.93	0.18	212.47	28.24
MN15-L	CPCM	18.10	3.00	0.20	249.97	3.19
N12	SMD	20.35	5.25	0.35	179.30	28.24
N12	CPCM	21.15	6.05	0.40	198.41	5.09
N12-SX	SMD	21.48	6.38	0.42	246.55	9.15
N12-SX	CPCM	22.51	7.41	0.49	293.64	9.40
Wb97XD	SMD	20.32	5.22	0.35	171.59	25.93
Wb97XD	CPCM	19.77	4.67	0.31	227.76	6.27

[a] Activation-free energies for reactions of AFB1 exo-8.9-epoxide with guanine calculated with 14 DFT functionals in conjunction with SMD and CPCM solvation models. [b] The absolute error between calculated and experimental activation-free energies for reactions between AFB1 exo-8.9-epoxide and guanine. [c]The relative error between calculated and experimental activation-free energies for reactions between AFB1 exo-8.9-epoxide and guanine. [d] The exactly one imaginary vibrational frequency of the transition state structure. [e] The lowest vibrational frequency of the reactant state structure.

Table S10. The average absolute and relative errors for the reactions between nine investigated chemical carcinogens and guanine calculated with 14 tested DFT functionals in conjunction with SMD and CPCM solvation models.

Functional	Solvation model	$\overline{\lvert \Delta(\Delta G) \rvert}$ [kcal/mol][a]	$\frac{\overline{\lvert \Delta(\Delta G) \rvert}}{\Delta G^{\ddagger}_{exp}}$ [/][b]
M11-L	SMD	1.08	0.05
MN12-L	SMD	1.11	0.05
N12-SX	SMD	2.28	0.12
M06-HF	SMD	2.39	0.13
WB97XD	SMD	2.41	0.12
M05-2X	SMD	2.5	0.13
B3LYP-D3	CPCM	2.61	0.13
MN12-L	CPCM	2.72	0.13
N12	SMD	2.73	0.14
MN12-SX	SMD	2.8	0.14
MN15-L	SMD	2.88	0.13
MN15	SMD	3.08	0.16
N12	CPCM	3.23	0.16
M08-HX	SMD	3.33	0.17
M11-L	CPCM	3.36	0.17
B3LYP-D3	SMD	3.43	0.17
N12-SX	CPCM	3.53	0.18
WB97XD	CPCM	3.59	0.17
M05-2X	CPCM	3.73	0.18
M11	SMD	3.78	0.18
M06-HF	CPCM	3.78	0.18
MN15-L	CPCM	3.88	0.18
MN15	CPCM	3.89	0.19
MN12-SX	CPCM	4.05	0.20
M06-2X	SMD	4.23	0.21
M08-HX	CPCM	4.28	0.21
M11	CPCM	5.03	0.24
M06-2X	CPCM	5.26	0.25

[a]Average absolute errors for the reactions between nine investigated chemical carcinogens and guanine calculated with 14 DFT functionals in conjunction with SMD and CPCM solvation models.
[b]The average relative errors for the reactions between nine investigated chemical carcinogens and guanine calculated with 14 DFT functionals in conjunction with SMD and CPCM solvation models.

Table S11. Computational results obtained with MN12-L functional in conjunction with 6-311++G(d,p) flexible basis set and SMD implicit solvation model for the reactions of nine investigated chemical carcinogens with EGCG, EGC, (+)-catechin, and glutathione.

Functional /solvation model M11-L/SMD	$\Delta G^{\ddagger}_{SMD}$ [kcal/mol][a]	ω^{TS} [i cm^{-1}][b]	ω^{R} [cm^{-1}][c]	d^{TS} [Å][d]	d^{R} [Å][e]	$r^{glutathione}$ [/][f]	$\Delta G^{\ddagger}_{guanine}$ [kcal/mol][g]
**Styrene Oxide**							
**EGCG**	23.49	-541.36	8.41	1.99	4.55	1.69	25.50
**EGC**	17.80	-531.27	13.21	2.03	4.84	1.28	25.50
**(+)-Catechin**	12.52	-539.47	7.11	2.04	3.89	0.90	25.50
**Glutathione**	13.87	-524.06	15.68	2.57	3.49	1.00	25.50
**Propylene Oxide**							
**EGCG**	26.07	-533.32	16.41	2.00	3.18	1.73	25.45
**EGC**	22.77	-530.16	23.45	2.04	3.71	1.51	25.45
**(+)-Catechin**	18.19	-521.91	16.04	2.04	3.40	1.21	25.45
**Glutathione**	15.03	-508.23	17.06	2.57	3.53	1.00	25.45
**Ethylene Oxide**							
**EGCG**	20.28	-546.99	8.57	2.03	4.58	1.18	23.29
**EGC**	19.65	-517.00	4.98	2.06	4.68	1.14	23.29
**(+)-Catechin**	19.37	-528.55	9.00	2.06	3.36	1.12	23.29
**Glutathione**	17.25	-534.01	10.74	2.54	4.99	1.00	23.29
**Glycidamide**							
**EGCG**	15.82	-527.85	15.03	2.11	3.38	1.45	23.35
**EGC**	10.19	-519.72	10.54	2.15	3.71	0.93	23.35
**(+)-Catechin**	10.18	-527.13	13.53	2.13	3.75	0.93	23.35
**Glutathione**	10.92	-504.68	10.74	2.63	4.70	1.00	23.35
**Vinyl Carbamate Epoxide**							
**EGCG**	19.25	-568.59	5.82	2.09	3.10	1.59	20.95
**EGC**	11.86	-576.95	21.11	2.12	3.11	0.98	20.95
**(+)-Catechin**	10.84	-587.48	18.23	2.10	3.20	0.89	20.95
**Glutathione**	12.13	-562.47	23.48	2.62	3.82	1.00	20.95
**Beta Propiolactone**							
**EGCG**	16.08	-533.32	8.59	2.13	3.66	2.36	21.34
**EGC**	9.53	-533.78	11.52	2.17	3.40	1.40	21.34
**(+)-Catechin**	6.35	-534.22	12.11	2.16	3.33	0.93	21.34
**Glutathione**	6.81	-524.98	28.28	2.71	3.40	1.00	21.34
**Chloroethylene Oxide**							
**EGCG**	17.59	-556.25	14.12	2.04	3.22	1.39	17.64
**EGC**	16.48	-554.64	9.75	2.07	3.26	1.30	17.64
**(+)-Catechin**	13.62	-561.69	13.55	2.06	3.26	1.07	17.64
**Glutathione**	12.64	-550.56	8.83	2.62	3.68	1.00	17.64

			**2-Cyanoethylene Oxide**				
**EGCG**	20.01	-612.51	6.30	2.04	3.30	1.55	20.57
**EGC**	17.97	-634.52	5.88	2.07	3.37	1.39	20.57
**(+)-Catechin**	13.95	-644.96	3.85	2.05	3.45	1.08	20.57
**Glutathione**	12.92	-712.15	9.04	2.63	3.68	1.00	20.57
			**AFB1 Exo-8.9-Epoxide**				
**EGCG**	16.43	-233.66	10.85	2.24	5.64	1.38	16.88
**EGC**	16.77	-280.43	21.90	2.33	3.18	1.40	16.88
**(+)-Catechin**	12.33	-175.97	17.07	2.28	3.15	1.03	16.88
**Glutathione**	11.94	-251.47	11.08	2.89	4.54	1.00	16.88

[a] Activation-free energies obtained with the functional MN12-L in combination with 6-311++G(d,p) flexible basis set and SMD solvation model. [b] The exactly one imaginary vibrational frequency of the transition state structure. [c] The lowest vibrational frequency of the reactant state structure. [d] The distance in the transition state structure between the most nucleophilic phenolic oxygen of EGCG, EGC, (+)-catechin or sulfur atom of glutathione, and the achiral electrophilic epoxy carbon of the chemical carcinogen. [e] The distance in the reactant structure between the most nucleophilic phenolic oxygen of EGCG, EGC, (+)-catechin or sulfur atom of glutathione and the achiral electrophilic epoxy carbon of the chemical carcinogen. [f] The ratio of activation-free energies between the investigated polyphenols, namely EGCG, EGC, (+)-catechin, and glutathione allowing the determination of relative carcinogen scavenging activity. [g] Activation-free energy of guanine calculated with the functional MN12-L in combination with 6-311++G(d,p) flexible basis set and SMD solvation model.
